# Human Leukocyte Antigen Complex and Other Immunogenetic and Clinical Factors Influence Susceptibility or Protection to SARS-CoV-2 Infection and Severity of the Disease Course. The Sardinian Experience

**DOI:** 10.3389/fimmu.2020.605688

**Published:** 2020-12-04

**Authors:** Roberto Littera, Marcello Campagna, Silvia Deidda, Goffredo Angioni, Selene Cipri, Maurizio Melis, Davide Firinu, Simonetta Santus, Alberto Lai, Rita Porcella, Sara Lai, Stefania Rassu, Rosetta Scioscia, Federico Meloni, Daniele Schirru, William Cordeddu, Marta Anna Kowalik, Maria Serra, Paola Ragatzu, Mauro Giovanni Carta, Stefano Del Giacco, Angelo Restivo, Simona Deidda, Sandro Orrù, Antonella Palimodde, Roberto Perra, Germano Orrù, Maria Conti, Cinzia Balestrieri, Giancarlo Serra, Simona Onali, Francesco Marongiu, Andrea Perra, Luchino Chessa

**Affiliations:** ^1^ Complex Structure of Medical Genetics, R. Binaghi Hospital, Area Socio-Sanitaria Locale (ASSL) Cagliari, Azienda per la Tutela della Salute (ATS) Sardegna, Italy; ^2^ Associazione per l’Avanzamento della Ricerca per i Trapianti O.d.V., non profit organisation, Cagliari, Italy; ^3^ Department of Medical Sciences and Public Health, University of Cagliari, Cagliari, Italy; ^4^ Complex Structure of Pneumology, PO SS Trinità, ASSL Cagliari, ATS Sardegna, Cagliari, Italy; ^5^ Complex Structure of Infectious Diseases, PO SS Trinità, ASSL Cagliari ATS Sardegna, Cagliari, Italy; ^6^ Department of Medical Biotechnology and Translational Medicine, University of Milan, Milan, Italy; ^7^ Unitá di Crisi Locale (UCL) ATS Sardegna, Cagliari, Italy; ^8^ Unit of Oncology and Molecular Pathology, Department of Biomedical Sciences, University of Cagliari, Cagliari, Italy; ^9^ Medical Genetics, Department of Medical Sciences and Public Health, University of Cagliari, Cagliari, Italy; ^10^ Colorectal Surgery Unit, Department of Surgical Science, University of Cagliari, Cagliari, Italy; ^11^ Molecular Biology Service Laboratory, Department of Surgical Science, University of Cagliari, Cagliari, Italy; ^12^ Liver Unit, Department of Internal Medicine, University Hospital of Cagliari, Cagliari, Italy

**Keywords:** human leukocyte antigen, SARS-CoV-2 infection, immunogenetic background, COVID-19 severity, Sardinian population, alleles, haplotypes, glucose-6-phosphate dehydrogenase

## Abstract

**Aim:**

SARS-CoV-2 infection is a world-wide public health problem. Several aspects of its pathogenesis and the related clinical consequences still need elucidation. In Italy, Sardinia has had very low numbers of infections. Taking advantage of the low genetic polymorphism in the Sardinian population, we analyzed clinical, genetic and immunogenetic factors, with particular attention to HLA class I and II molecules, to evaluate their influence on susceptibility to SARS-CoV-2 infection and the clinical outcome.

**Method and Materials:**

We recruited 619 healthy Sardinian controls and 182 SARS-CoV-2 patients. Thirty-nine patients required hospital care and 143 were without symptoms, pauci-symptomatic or with mild disease. For all participants, we collected demographic and clinical data and analyzed the HLA allele and haplotype frequencies.

**Results:**

Male sex and older age were more frequent in hospitalized patients, none of whom had been vaccinated during the previous seasonal flu vaccination campaignes. Compared to the group of asymptomatic or pauci-symptomatic patients, hospitalized patients also had a higher frequency of autoimmune diseases and glucose-6-phosphate-dehydrogenase (G6PDH) deficiency. None of these patients carried the beta-thalassemia trait, a relatively common finding in the Sardinian population. The extended haplotype HLA-A*02:05, B*58:01, C*07:01, DRB1*03:01 [OR 0.1 (95% CI 0–0.6), Pc = 0.015] was absent in all 182 patients, while the HLA-C*04:01 allele and the three-loci haplotype HLA-A*30:02, B*14:02, C*08:02 [OR 3.8 (95% CI 1.8–8.1), Pc = 0.025] were more frequently represented in patients than controls. In a comparison between in-patients and home care patients, the HLA-DRB1*08:01 allele was exclusively present in the hospitalized patients [OR > 2.5 (95% CI 2.7–220.6), Pc = 0.024].

**Conclusion:**

The data emerging from our study suggest that the extended haplotype HLA-A*02:05, B*58:01, C*07:01, DRB1*03:01 has a protective effect against SARS-CoV-2 infection in the Sardinian population. Genetic factors that resulted to have a negative influence on the disease course were presence of the HLA-DRB1*08:01 allele and G6PDH deficiency, but not the beta-thalassemic trait. Absence of influenza vaccination could be a predisposing factor for more severe disease.

## Introduction

Outbreak of the coronavirus disease (COVID-19) was declared a pandemic by the World Health Organization (WHO) on 11 March 2020 (1) and since then continues to pose a threat to the sustainability of public health systems worldwide. Although consensus on total mortality and morbidity has yet to be reached, current data show that over 50 million people have contracted the SARS-CoV-2 virus globally, and of these, more than 1,250,000 have died (2).

Although only a few months have passed since the onset of the pandemic, a myriad of attempts have been made to unravel the unknowns surrounding disease pathogenesis and the related clinical consequences. Currently, it is assumed that the mean incubation period is 4–6 days with an estimated range of 2–14 days, although rare cases of up to 27 days have been reported. The large majority of patients have a good prognosis with an asymptomatic or pauci-symptomatic disease course (3). However, about 20% of infected subjects become seriously ill and 4% develop severe and even fatal disease. Clinical features in hospitalized patients include complications such as pneumonia and, in the most severe cases, acute respiratory distress syndrome (ARDS). Devastating neurological and gastrointestinal symptoms have also been reported. Sepsis is another feared complication of COVID-19 that can cause lasting damage to the lungs and other organs. Moreover, heart failure, kidney failure, liver damage, hypercoagulability, septic shock, and multiple-organ failure (MOF) have been shown to precipitate death. The most severe cases of COVID-19 with admission to the Intensive Care Unit (ICU) are generally more frequent in males and the elderly, especially those with comorbidities such as chronic cardiovascular and/or respiratory disease (4–8).

A plethora of drugs and treatments have been tried around the world in the battle against the pandemic but, so far, none of those tested or included in clinical trials have yielded a significant reduction in the morbidity and mortality rates (9). Contemporarily, the scientific community is striving hard to produce a vaccine but in spite of the many encouraging results, health experts warn that a coronavirus vaccine is unlikely to be available in time to stop the second wave of the epidemic which is expected to peak in European countries, including Italy, this autumn or winter (10).

After China and South Korea, Italy was the first European country to be hit by the explosive pandemic potential of COVID-19, which sparked from the first cases diagnosed in the province of Lodi (11) to a quickly rising toll of 250,000 infected individuals and more than 35,000 deaths. The Northern regions of Italy were the territories with the large majority of cases (12). A decreasing gradient of infection levels from north to south was clearly evident from the beginning of the epidemic, with atypical differences even between the provinces of the most affected regions (13).

Data published by the Italian National Institute of Statistics (ISTAT) and the Italian National Institute of Health (ISS) on 3 August 2020 demonstrated that the number of people who had been infected with the virus was six-fold higher than the total number of individuals who had been identified by positive test results for both nasopharyngeal and oropharyngeal swabs. The seroprevalence survey on SARS-CoV-2 conducted at national level showed that 1 million 482,000 individuals tested positive for anti-SARS-CoV-2 IgG antibodies, corresponding to 2.5% of the resident population. Data analysis confirmed the important differences observed for spread of the infection between the northern and southern regions of Italy, with the Islands of Sardinia and Sicily having the lowest seroprevalence of anti-SARS-CoV-2 IgG antibodies (0.3%) (14). The different routes of transmission as well as the clinical, epidemiological and virologic features of COVID-19 still need to be clarified today, at more than 6 months from the start of the pandemic (15, 16).

While some traditional or home remedies may alleviate symptoms of mild COVID-19, there are still no drugs or treatments that have been shown to prevent or cure the disease. An initially high viral load has been shown to be independently associated with disease severity and could be influenced by host immune responses (17). Based on the experience gained during the previous Severe Acute Respiratory Syndrome (SARS) and Middle East Respiratory Syndrome (MERS) epidemics, it is likely that both innate and adaptive host immunity play a role in viral clearance, disease severity and the different clinical manifestations of the disease (18).

Recently published data suggest that appropriate innate and adaptive T-cell-mediated humoral and cellular immune responses could have a critical role in the elimination of the SARS-CoV-2 virus and confer consequent protective immunity, which in most cases coincides with clinical recovery (11, 19, 20). On the other hand, an excessive cell-mediated and dysregulated innate and adaptive immune response can lead to an aggressive inflammatory reaction with the release of large amounts of pro-inflammatory cytokines. This so-called “cytokine storm”—resulting from the excessive production of cytokines by immune cells such as the innate dendritic cells, macrophages, natural killer (NK) cells and the adaptive T and B cells—directly correlates with lung injury, ARDS and MOF including the kidneys and the central nervous system and overall leads to an unfavourable prognosis (5, 11, 21–26).

The human leukocyte antigen (HLA) class I and II molecules are actively engaged in immune response mechanisms against invading pathogens. Their pivotal role in viral immune response has already been highlighted in studies performed on the two coronaviruses responsible for SARS and MERS. Also, in SARS-Cov-2 infection, there is evidence of how different HLA alleles of the major histocompatibility complex (MHC) can define individual susceptibility to infection and its spread (19).

Previous studies on different viruses have shown a correlation between the susceptibility and/or severity of disease and the genetic variability of HLA alleles. These alleles are critical components of the viral antigen presentation pathway. In fact, a study carried out on transgenic mice has shown that the DRB1*04:01 allele, which confers susceptibility to certain autoimmune diseases, generates a robust toll-like receptor (TLR) response and eliminates H1N1 infection. Furthermore, following vaccination and exposure to the H1N1 influenza strain, these mice exhibited cross protective immunity also for the H3N2 strain (27).

Interesting studies conducted on the SARS-CoV-1 virus have identified HLA polymorphisms associated with the disease risk in the Asian population. The HLA-B*46:01 allele was observed with a high frequency in a group of patients from Taiwan classified as being “probable SARS cases” from coronavirus infection. This allele was also significantly increased in the group of seriously ill patients (28). In Chinese patients, the HLA-B*07:03 and HLA-DRB1*03:01 alleles closely correlated with SARS-CoV-1 infection. Moreover, the HLA-B*07:03 and HLA-B*60 alleles were observed with a significantly higher frequency in patients in comparison to the expected frequency of the general population (29). In a group of SARS-CoV-1 positive Vietnamese patients, a strong association was observed for HLA-DRB1*12:02 in comparison to the control group (30). Also, the HLA-C*08:01 allele seemed to confer susceptibility to SARS-CoV-1 (31).

Immunogenetic variation in humans is becoming an increasingly important target for clinical diagnosis and therapeutic intervention. Binding between peptide epitopes and HLA proteins significantly contributes to cellular immune response mechanisms in human beings. Several studies describe the pivotal role of peptides in the specificity, magnitude and quality of both humoral and cellular immune responses. This is furthermore supported by the recent emphasis put on the use of peptides in vaccine design and medical diagnostics. In silico studies have greatly facilitated analysis of the binding affinity between all the viral peptides of SARS-CoV-2 and different HLA class I genotypes. It has been shown that the HLA-B*46:01 allele has a low degree of binding affinity, suggesting that subjects with this allele may have a higher risk of developing the more severe forms of COVID-19. On the other hand, the HLA-B*15:03 allele is reported as having the highest binding affinity for viral peptides (32).

Another study describes high binding affinity between SARS-CoV-2 epitopes and the HLA-A*02:06, HLA-B*52:01, and HLA-C*12:02 alleles. In particular, two epitopes displayed strong binding affinity for HLA-A*24:02, HLA-A*02:01, and HLA-A*02:06 (33). Unfortunately, these two studies present mathematical predictions that need to be clinically and experimentally evaluated and confirmed in a suitable study population with appropriate immunogenetic characteristics.

Researchers in Italy set up a study to investigate whether specific class I HLA alleles could at least partially explain the huge differences observed for the spread of SARS-CoV-2 infection between northern and southern Italy. They compared HLA allele prevalence retrieved through the Italian Bone-Marrow Donor Registry with the incidence of SARS-CoV-2 infections in the different geographical regions. It emerged that HLA-A*25, B*08, B*44, B*15:01, B*51, C*01, and C*03 positively correlated with the incidence of SARS-CoV-2 infection, while HLA-B*14, B*18, and B*49 showed an inverse correlation. After applying a multiple regression model to eliminate confounding factors, only the HLA-C*01 and –B*44 alleles, which are present with a higher frequency in the northern regions of Italy, remained positively associated with COVID-19 (34).

Sardinia is an autonomous region of Italy and the second-largest Island in the Mediterranean Sea. The Island has one of the lowest infection and mortality rates in Italy and on 7 November 2020 registered 11,412 positive individuals (infections: 7x10^-3^ Sardinian inhabitants vs 14x10^-3^ Italian mainland inhabitants) and 249 deaths (mortality: 2.18% Sardinian inhabitants vs 3.81% Italian mainland inhabitants). Moreover, during the second wave of the epidemic, Sardinia continued to be one of the Italian regions with the lowest transmission rate (Rt) for SARS-CoV-2 (Sardinian Rt index = 1.16 vs Italian Rt index 1.7— http://www.salute.gov.it/portale/nuovocoronavirus/).

The geographic isolation of Sardinia throughout history has limited the genetic diversity of its people. The population with its low levels of genetic polymorphism has made important contributions to genome-wide association studies of complex disease traits. Many disease risk alleles are shared between Sardinian and other populations which makes it an ideal location for the identification of immunogenetic factors potentially involved in resistance or susceptibility to SARS-CoV-2 infection.

Based on these premises, we set up a study to investigate the clinical characteristics and genetic traits of Sardinian patients affected by SARS-CoV-2 infection. Particular emphasis was put on the influence of HLA Class I and II alleles on disease susceptibility and the varying severity of the disease course.

## Materials and Methods

A cohort of 182 patients were recruited from 1 June to 1 August 2020. All patients had been diagnosed with SARS-CoV-2 by RT-PCR from nasopharyngeal swab. The patients were divided into two groups according to disease severity. Thirty-nine patients had been admitted to the Covid Unit of the SS.Trinità Hospital in Cagliari with moderate or severe disease (group S) and 143 asymptomatic or pauci-symptomatic patients (group A) were confined to home quarantine. According to WHO classification, patients with severe disease were considered to be those who needed invasive mechanical ventilation or high-flow nasal oxygen in the hospital, while patients classified as having moderate symptoms did not require oxygen. Pauci-symptomatic patients presented mild symptoms such as loss of taste or smell and/or cold or flu-like symptoms (35). Six hundred and nineteen healthy controls, without SARS-CoV-2 infection (negative for RT-PCR from nasopharyngeal swab), were extracted from the Sardinian Voluntary Bone Marrow Donor Registry, which is highly representative of the genetically homogeneous island population of Sardinia, Italy (36, 37). The controls were selected to appropriately represent the male-to-female ratio and genetic frequencies of the population pertaining to the areas of central and southern Sardinia from where the COVID-19 patients were recruited.

The HLA class I and class II allele frequencies (HLA-A, -B, -C, -DRB1) observed in the two groups of patients (Group S and Group A) were compared to those of the 619 unrelated healthy controls.

### Ethics Statement

Patients were recruited and enrolled in the study protocol at the Department of Medical Sciences and Public Health of the University of Cagliari, the University Hospital of Cagliari (AOUCA) and the SS.Trinità Hospital of the Sardinian Regional Company for the Protection of Health (ATS Sardegna). Written informed consent was obtained from all patients and controls in accordance with the ethical standards (institutional and national) of the local human research committee. The study protocol, including informed consent procedures, conforms to the ethical guidelines of the Declaration of Helsinki and was approved by the responsible ethics committee (Ethics Committee of the Cagliari University Hospital; date of approval: May, 27, 2020; protocol number GT/2020/10894). Records of written informed consent are kept on file and are included in the clinical record of each patient.

### HLA Allele Typing

DNA from nasopharyngeal swab was extracted with the Qiagen QIAamp DNA Mini Kit (Qiagen, Valencia, CA, USA) according to the manufacturer’s instructions. Briefly, each swab was washed with PBS (200 µl). The recovered washing buffer was mixed with 20 µl of proteinase k and 200 µl of the AL buffer was added to the mix. After 10 min of incubation at 56°C, the sample was processed according to the kit instructions. Purity and quantification of total DNA were assessed with the NanoDrop 1000 Spectrophotometer (Thermo Fisher Scientific).

Patients and controls were typed at high resolution for the alleles at the HLA-A, B, C, and DRB1 loci using a polymerase chain reaction sequence-speciﬁc primer (PCR-SSP) method according to the manufacturer’s instructions (Allele-speciﬁc PCR-SSP kits: Olerup SSP AB, Stockholm, Sweden).

HLA typing validation was performed by Next Generation Sequencing on 96 randomly selected samples of patients and controls, according to a previously reported method (38). The concordance of the two different methods was 100%.

### HLA Haplotype Analysis in Patients and Controls

Four-loci HLA haplotypes are sequences of four HLA alleles localized in four different HLA loci: HLA-A*a_i_, HLA-B*b_j_, HLA-C*c_h_, HLA-DRB1*d_k_, where *a_i_*, *b_j_*, *c_h_*, *d_k_* are integers (or couples of integers in high resolution) and each index *i*, *j*, *h*, *k* = 1,2 indicates one of the two alleles in the respective HLA-locus. The four-loci HLA haplotypes in each subject are 2^4^ = 16, obtained by combining four alleles localized in four different HLA loci. Analogously, the three-loci HLA haplotypes in each subject are 2^3^ = 8, obtained by combining three alleles localized in three different HLA loci. Finally, the two-loci HLA haplotypes are 2^2^ = 4, formed by two alleles localized in two different HLA loci.

The frequency of each HLA haplotype in a sample of size is computed by dividing the number of times the HLA haplotype appears in that sample by the total number of alleles for each HLA locus.

The HLA analysis in patients and controls was performed using a specific programming code written with R language (R core39). The statistically significant outcomes given by the R code were also checked “by hand” through the direct evaluation of the number of HLA haplotypes in each group of subjects.

### Statistical Analysis

The clinical and genetic characteristics of patients with SARS-CoV-2 infection were analyzed using R software version 4.0.2 (39). Mean standard deviation (SD) and interquartile range (IQR) were calculated for all continuous variables; percentages and 95% confidence intervals (95% CI) were computed for categorical data. P values were calculated using two-tailed Fisher’s exact test or Student’s test as appropriate. Only P values below 0.05 were considered to be statistically significant.

To compare the HLA allele and haplotype frequencies of COVID-19 patients with those of the healthy controls we used the two-tailed Fisher’s exact test. For each single HLA allele, Pc was obtained by multiplying the uncorrected P value by the number of alleles observed. The frequencies of the HLA extended (four loci) and partial (three or two loci) haplotypes were obtained analytically using a programming code created with R language.

Cochran’s rule—claiming that a minimum expected frequency of 5 can be regarded as adequate in analyzing tables with more than a single degree of freedom—was used to establish which HLA haplotypes had frequencies sufficiently high to allow for significant comparisons between the two groups of patients and controls.

For each HLA haplotype the corrected P value (Pc) was calculated by multiplying the P value obtained with the two-tailed Fisher’s exact test by the number of tested allelic combinations. Only Pc values lower than 0.05 were considered to be statistically significant (see **Supplementary Material**).

## Results

### Main Clinical and Genetic Characteristics of Sardinian COVID-19 Patients

The most relevant clinical and genetic features of patients positive for SARS-CoV-2 are shown in **Table 1**. Mean age at the time of infection was 53 years (mean ± SD: 53.2 ± 18.1; 95% CI 50.6–55.9; IQR = 27.4), with a prevalence of female patients (61.5%). Moreover, 53% (n = 96) of the patients had an age at onset of ≤ 50 years; 21% (n = 38) of the patients were over 65 years of age. In line with previous clinical studies, the most severe symptoms were present in adults aged 65 years and older [OR 10.1 (95% CI 4.2–25.2), P = 1.5 10^-8^]. The clinical manifestations were less severe in female patients (n =112). In fact, 28 of the 39 critically ill patients were males [71.8% vs 28.2%, OR 6.1 (95% CI 2.6–14.8), P = 2.2 10^-6^].

**Table 1:** Comparisons of baseline clinical, genetic and biochemical parameters between COVID-19 patients with asymptomatic/pauci-symptomatic and moderate/severe disease.

Characteristics of Sardinian COVID-19 pts	Total pts (N = 182)		Group A (N = 143)		Group S (N = 39)		Comparison Group S vs Group A	
Age (yr): mean ± SD (95% CI; IQR)	53.2 ± 18.1 (50.6–55.9; 27.4)		49.1 ± 17.2 (46.3–52.0; 20.9)		66.1 ± 15.3 (61.1–71.0; 26.8)		P = 9.2 · 10^-8^	
	**n (%)**	**95% CI (%)**	**n (%)**	**95% CI**	**n (%)**	**95% CI (%)**	**P value**	**OR (95% CI)**
Age ≤ 50 yr	96 (52.7)	45.4–60.0	85 (59.4)	51.3–67.6	11 (28.2)	13.6–42.8	5.8 10^-4^	0.3 (0.1–0.6)
50 yr < Age < 65 yr	48 (26.4)	19.9–32.8	42 (29.4)	21.8–36.9	6 (15.4)	3.7–27.1	0.101	0.4 (0.1–1.2)
Age ≥ 65 yr	38 (20.9)	14.9–26.8	16 (11.2)	6.0–16.4	22 (56.4)	40.3–72.5	1.5 10^-8^	10.1 (4.2–25.2)
Male	70 (38.5)	31.3–45.6	42 (29.4)	21.8–36.9	28 (71.8)	57.2–86.4	2.2 10^-6^	6.1 (2.6–14.8)
Female	112 (61.5)	54.4–68.7	101 (70.6)	63.1–78.2	11 (28.2)	13.6–42.8	2.2 10^-6^	0.2 (0.1–0.4)
FLU vaccine 2019 (total pts)	23 (12.6)	7.8–17.5	23 (16.1)	10.0–22.2	0	0.0–9.0	0.005	0.0 (0.0–0.6)
- FLU vaccine 2019 (age ≥ 65 yr)	0	0.0–2.0	0	0.0–2.5	0	0.0–9.0	1	–
FLU vaccine last 3 yr	27 (14.8)	9.6–20.0	27 (18.9)	12.4–25.4	0	0.0–9.0	0.002	0.0 (0.0–0.5)
- FLU vaccine last 3 yr (age ≥ 65 yr)	0	0.0–2.0	0	0.0–2.5	0	0.0–9.0	1	–
***Comorbidity***								
Cancer	4 (2.2)	0.1–4.3	4 (2.8)	0.1–5.5	0	0.0–9.0	0.579	0.0 (0.0–5.6)
Type I Diabetes Mellitus	6 (3.3)	0.7–5.9	4 (2.8)	0.1–5.5	2 (5.1)	0.0–12.3	0.610	1.9 (0.2–13.6)
Chronic pulmonary disease^1^	2 (1.1)	0.0–2.6	2 (1.4)	0.0–3.3	0	0.0–9.0	1	0.0 (0.0–19.7)
Ischemic heart disease^2^	14 (7.7)	3.8–11.6	12 (8.4)	3.8–13.0	2 (5.1)	0.0–12.3	0.737	0.6 (0.1–2.8)
Hypertension	27 (14.8)	9.6–20.0	21 (14.7)	8.8–20.5	6 (15.4)	3.7–27.1	1	1.1 (0.3–3.0)
Autoimmune disease^3^	22 (12.1)	7.3–16.9	11 (7.7)	3.3–12.1	11 (28.2)	13.6–42.8	0.001	4.7 (1.7–13.2)
Hypercholesterolemia	18 (9.9)	5.5–14.3	11 (7.7)	3.3–12.1	7 (17.9)	5.5–30.4	0.071	2.6 (0.8–8.1)
***Chronic Medication use***								
Steroidal anti-inflammatory drug	9 (4.9)	1.8–8.1	6 (4.2)	0.9–7.5	3 (7.7)	0.0–16.3	0.406	1.9 (0.3–9.4)
Non-steroidal anti-inflammatory drug^4^	11 (6.0)	2.6–9.5	6 (4.2)	0.9–7.5	5 (12.8)	2.0–23.7	0.060	3.3 (0.8–14.0)
ACE II inhibitor^5^	19 (10.4)	6.0–14.9	12 (8.4)	3.8–13.0	7 (17.9)	5.5–30.4	0.134	2.4 (0.7–7.2)
Angiotensin II receptor blocker^6^	10 (5.5)	2.2–8.8	7 (4.9)	1.3–8.5	3 (7.7)	0.0–16.3	0.449	1.6 (0.3–7.5)
Beta and calcium channel blockers^7^	25 (13.7)	8.7–18.8	16 (11.2)	6.0–16.4	9 (23.1)	9.4–36.7	0.068	2.4 (0.8–6.4)
Levothyroxine	10 (5.5)	2.2–8.8	10 (7.0)	2.8–11.2	0	0.0–9.0	0.122	0.0 (0.0–1.6)
***Genetic trait***								
Beta-thalassemic Trait	19 (10.4)	6.0–14.9	19 (13.3)	7.7–18.9	0	0.0–2.1	0.015	0.0 (0.0–0.7)
G6PDH deficiency	24 (13.2)	8.2–18.1	14 (9.8)	4.9–14.7	10 (25.6)	11.5–39.8	0.015	3.2 (1.1–8.5)
***Serology***	**Mean ± SD**	**95% CI (IQR)**	**Mean ± SD**	**95% CI (IQR)**	**Mean ± SD**	**95% CI (IQR)**	**P value**	
White blood cell count (x10^3^/µL)	8.3 ± 3.2	7.8–8.7 (4.5)	8.1 ± 2.3	7.7–8.5 (4.5)	8.4 ± 3.7	7.3–9.6 (4.6)	0.444	
Lymphocyte count (x10^3^/µL)	1.1 ± 0.7	1.0–1.2 (0.5)	1.2 ± 0.5	1.1–1.2 (0.7)	1.0 ± 0.8	0.8–1.3 (0.6)	0.182	

Group A: patients with asymptomatic or pauci-symptomatic disease.

Group S: moderate or severe disease.

SD, Standard deviation; IQR, Interquartile range; CI, Confidence interval.

^1^Chronic obstructive pulmonary disease was defined as a diagnosis of emphysema and/or bronchitis.

^2^Ischemic heart disease was categorized as a history of myocardial infarction or angina.

^3^Autoimmune disease included Hashimoto’s thyroiditis, type I diabetes mellitus, rheumatoid arthritis, systemic lupus erythematosus, and autoimmune hepatitis.

^4^Non-steroidal anti-inflammatory drugs included aspirin, ibuprofen, diclofenac, naproxen, indomethacin, celecoxib, and meloxicam.

^5^Angiotensin-converting enzyme II inhibitors included captopril, enalapril, lisinopril, fosinopril, ramipril, and quinapril.

^6^Angiotensin II receptor blockers included losartan, candesartan, irbesartan, olmesartan, and valsartan.

^7^Dihydropyridine calcium channel blockers included amlodipine and nifedipine. Beta blockers included atenolol, bisoprolol, labetalol, metoprolol, and nebivolol.

Approximately 13% of the examined subjects had been vaccinated against the flu in the last year. This percentage rose to 15% when the 3-year period 2017-2020 was considered. None of the 39 patients with moderate or severe disease had received anti-flu vaccination during this 3-year period, unlike the group of patients with pauci-symptomatic infection or mild symptoms [0% vs 16.1%, OR 0.07 (95% CI 0–0.5), P = 0.002]. During the winter season in Sardinia of 2019, about 14.2% of the general population underwent flu vaccination. An annual flu vaccination is strongly recommended for people at risk of severe outcomes and in 2019 was administered to 46,5% of the population over 65 years of age (40). It is interesting to note that none of the 22 seriously ill patients, who were over the age of 65, had received a flu vaccination in recent years.

About 8% of the subjects examined had ischemic heart disease, 15% arterial hypertension, 10% hypercholesterolemia, 12% autoimmune diseases and 3.3% type 1 diabetes mellitus. Comorbidities were present in both groups of patients. The only significant difference was a higher frequency of autoimmune diseases (rheumatoid arthritis, type I diabetes and autoimmune hepatitis) in group S [28.2% vs 7.7%, OR 4.7 (95% CI 1.9–11.9), P = 0.001]. Type I diabetes mellitus was present with a higher frequency in the group of patients with a severe clinical picture (5.1% vs 2.8%), but considered separately, did not reach statistical significance.

Five percent (5%) of group S subjects took steroidal anti-inflammatory drugs, 6% non-steroidal anti-inflammatory drugs, 10.4% ACE II inhibitors and/or blockers of angiotensin II receptors (5.5%), 13.7% beta blockers or calcium channel blockers. No statistically significant differences were observed when comparing chronic drug intake between the two groups.

Additionally, two very common genetic traits were considered in the Sardinian population: the beta thalassemic trait and the G6PDH enzyme deficiency. Nineteen COVID-19 positive patients (10.4%) were heterozygous for the β^0^-39 mutation of the beta globin chain. None of the group S patients carried this mutation [0% vs 13.3%, OR 0.2 (95% CI 0.1–0.8), P = 0.015].

Twenty-four patients (13.2%) had G6PDH enzyme deficiency, resulting in a markedly higher frequency in group S patients compared to group A patients [25.6% vs 9.8%, OR 3.2 (95% CI 1.3–7.9), P = 0.015]. The gene encoding G6PDH maps to the long arm of the X chromosome. There are different genetic variants of G6PDH depending on the geographical area. In Sardinia the enzyme deficiency is caused by the Mediterranean variant (G6PDH^Med^) which is characterized by the absence or marked reduction of enzymatic activity (0%–10% compared to normal enzyme activity) (41). It should be noted that all 10 patients (25.6%) with G6PDH deficiency of the 39 patients of group S were male and therefore lacked or had markedly reduced enzyme activity.

### Allele and Haplotype Frequencies Associated With SARS-CoV-2 Infection

The HLA allele frequencies are illustrated in **Table 2** and the **Supplementary Material**. The 364 alleles of the 182 patients infected by SARS-CoV-2 were compared to the 1,238 alleles of the 619 unrelated healthy controls representative of the HLA frequencies in the Sardinian population. No substantial differences were observed for the HLA Class I (HLA-A, HLA-B, HLA-C) and Class II (HLA-DRB1) alleles. Most of the significant differences (P value < 0.05) were lost after adjustment for multiple comparisons (Pc). The only allele that maintained significance was HLA-C*04:01 [OR = 1.8 (95% CI: 1.3–2.4), P = 0.001; Pc = 0.012]. The HLA-B40 antigen, which initially resulted to be significantly associated to SARS-Cov-2 (**Table 2**), only exhibited the single allele HLA-B*40:02 in the control group, while in patients it was present with two allelic variants: HLA-B*40:02 and HLA-B*40:01. Therefore, even if the HLA-B40 antigen remained weakly significant after correction for multiple-testing, the alleles lost statistical significance (P = 0.016, Pc = 0.24).

**Table 2:** Comparisons of HLA Class I and Class II allele frequencies between patients and controls and patients with an asymptomatic/pauci-symptomatic or moderate/severe disease course.

	Controls (1238 alleles)	Patients (364 alleles)	P values		Group A (286 alleles)		Group S (78 alleles)	P values	
	n (%)	n (%)	P	P_c_	n (%)		n (%)	P	P_c_
**HLA-A**									
A*11	81 (6.5)	36 (9.9)	0.039	0.694	32 (11.2)		6 (7.7)	0.530	1
A*26	28 (2.3)	14 (3.8)	0.133	1	7 (2.4)		2 (2.6)	1	1
A*30	230 (18.6)	56 (15.4)	0.186	1	45 (15.7)		12 (15.4)	1	1
A*03	65 (5.3)	26 (7.1)	0.197	1	19 (6.6)		4 (5.1)	0.795	1
A*33	45 (3.63)	8 (2.2)	0.242	1	7 (2.4)		2 (2.6)	1	1
A*29	26 (2.1)	4 (1.1)	0.274	1	4 (1.4)		0	0.582	1
A*32	108 (8.7)	38 (10.4)	0.351	1	28 (9.8)		10 (12.8)	0.411	1
A*01	102 (8.2)	24 (6.6)	0.375	1	21 (7.3)		2 (2.6)	0.187	1
A*02	363 (29.3)	98 (26.9)	0.392	1	79 (27.6)		23 (29.5)	0.778	1
A*24	119 (9.6)	38 (10.4)	0.618	1	32 (11.2)		6 (7.7)	0.530	1
A*23	20 (1.6)	4 (1.1)	0.626	1	0		4 (5.1)	0.002*	0.034
**HLA-B**									
B*40	11 (0.9)	12 (3.3)	0.001^†^	0.049	7 (2.4)		6 (7.7)	0.038	0.950
B*58	141 (11.9)	22 (6.0)	0.002	0.059	19 (6.6)		4 (5.1)	0.795	1
B*55	40 (3.2)	2 (0.5)	0.002	0.066	2 (0.7)		0	1	1
B*53	5 (0.4)	8 (2.2)	0.002	0.077	4 (1.4)		4 (5.1)	0.068	1
B*35	153 (12.4)	58 (15.9)	0.078	1	45 (15.7)		12 (15.4)	1	1
B*18	315 (25.4)	76 (20.9)	0.082	1	58 (20.3)		16 (20.5)	1	1
B*08	30 (2.4)	14 (3.8)	0.147	1	11 (3.8)		4 (5.1)	0.537	1
B*44	58 (4.7)	24 (6.6)	0.175	1	17 (5.9)		4 (5.1)	1	1
B*14	74 (6.0)	28 (7.7)	0.271	1	19 (6.6)		8 (10.3)	0.328	1
B*07	37 (3.0)	14 (3.8)	0.399	1	7 (2.4)		6 (7.7)	0.038	0.950
B*39	22 (1.8)	8 (2.2)	0.659	1	9 (3.1)		0	0.214	1
B*13	23 (1.9)	8 (2.2)	0.667	1	7 (2.4)		2 (2.6)	1	1
B*15	23 (1.9)	8 (2.2)	0.667	1	4 (1.4)		4 (5.1)	0.068	1
B*49	75 (6.1)	20 (5.5)	0.801	1	19 (6.6)		2 (2.6)	0.271	1
B*51	79 (6.4)	22 (6.0)	0.903	1	21 (7.3)		2 (2.6)	0.187	1
**HLA-C**									
C*04	139 (11.2)	66 (18.1)	0.001^	0.012	51 (17.8)		14 (17.9)	1	1
C*07	369 (29.8)	88 (24.2)	0.040	0.527	68 (23.8)		23 (29.5)	0.305	1
C*16	34 (2.7)	18 (4.9)	0.040	0.567	15 (5.2)		2 (2.6)	0.544	1
C*12	77 (6.2)	34 (9.3)	0.046	0.593	34 (11.9)		2 (2.6)	0.010	0.120
C*03	48 (3.9)	8 (2.2)	0.145	1	4 (1.4)		4 (5.1)	0.068	0.816
C*06	76 (6.1)	16 (4.4)	0.249	1	15 (5.2)		2 (2.6)	0.544	1
C*05	243 (19.6)	62 (17.0)	0.288	1	45 (15.7)		16 (20.5)	0.310	1
C*08	73 (5.9)	26 (7.1)	0.387	1	17 (5.9)		8 (10.3)	0.206	1
C*02	73 (5.9)	24 (6.6)	0.618	1	19 (6.6)		2 (2.6)	0.271	1
C*01	26 (2.1)	6 (1.6)	0.676	1	7 (2.4)		0	0.354	1
C*15	54 (4.4)	14 (3.8)	0.768	1	9 (3.1)		4 (5.1)	0.488	1
**HLA-DRB1**									
DR*14	34 (2.7)	20 (5.5)	0.0193	0.251	17	(5.9)	2 (2.6)	0.387	1
DR*11	195 (15.8)	44 (12.1)	0.0941	1	43	(15.0)	4 (5.1)	0.021	0.252
DR*12	19 (1.5)	2 (0.5)	0.193	1	2	(0.7)	0	1	1
DR*07	68 (5.5)	14 (3.8)	0.227	1	13	(4.5)	2 (2.6)	0.747	1
DR*13	51 (4.1)	18 (4.9)	0.466	1	17	(5.9)	2 (2.6)	0.387	1
DR*16	240 (19.4)	76 (20.9)	0.549	1	55	(19.2)	18 (23.1)	0.523	1
DR*01	104 (8.4)	34 (9.3)	0.595	1	23	(8.0)	10 (12.8)	0.189	1
DR*04	164 (13.2)	52 (14.3)	0.601	1	32	(11.2)	16 (20.5)	0.038	0.456
DR*03	272 (22.0)	82 (22.5)	0.829	1	66	(23.1)	16 (20.5)	0.760	1
DR*15	43 (3.5)	12 (3.3)	1	1	11	(3.8)	2 (2.6)	0.743	1
DR*08	23 (1.9)	4 (1.1)	0.486	1	0		4 (5.1)	0.002^#^	0.024

^HLA-C*04:01 was the only allelic variant observed for the HLA-C4 antigen. The difference observed for this allele between patients and controls remained significant after correction for multiple comparisons [OR = 1.8 (95% CI: 1.3–2.4)].

^†^For HLA-B40: HLA-B*40:02 was found in controls and HLA-B*40:02 and HLA-BB*40:01 in patients; statistical significance was lost after analyzing each single allele.

*The only allelic variant observed for HLA-A23 was HLA-A*23:01.

^#^The only allelic variant observed for HLA-DR8 was HLA-DRB1*08:01.

When comparing patients with pauci-symptomatic or mild disease to patients with moderate, severe or critical disease, only HLA-A*23:01 and HLA-DRB1*08:01 (OR > 2.5) maintained statistical significance after correction for multiple comparisons.

Group A: patients with asymptomatic or pauci-symptomatic disease.

Group S: patients with moderate or severe disease.

P_c_ was obtained by multiplying the uncorrected P value by the number of alleles observed.

More important results were obtained for the haplotypes that characterize the Sardinian population (**Table 3**). Rather surprisingly, none of the patients affected by COVID-19 carried the HLA-A*02:05, B*58:01, C*07:01, DRB1*03:01 extended haplotype [OR 0.1 (95% CI 0–0.6), P = 0.0006, Pc = 0.015]. After breaking down this extended haplotype into partial haplotypes with three or two loci, deriving from the different allelic combinations, it emerged that no patient infected with SARS-CoV-2 had the three-loci extended haplotype HLA-A*02:05, B*58:01, DR*03:01 [OR 0.1 (95% CI 0–0.5), P = 0.0007, Pc = 0.016]. Also, the HLA-A*02:05, B*58:01, C*07:01 three-loci haplotype had a significantly reduced frequency in patients compared to controls [OR 0.4 (95% CI 0.2–0.7), P = 0.0004, Pc = 0.011].

**Table 3:** Comparison of HLA haplotype frequencies between controls and patients.

HLA Haplotypes	Healthy controls (1238 haplotypes)	Covid-19 pts (364 haplotypes)			
	n (%)	n (%)	P	OR(95% CI)	P_c_
**Complete HLA haplotypes**					
HLA-A*02:05, B*58:01, C*07:01, DRB1*16:01	82 (6.6)	10 (2.7)	0.004	0.4 (0.2–0.8)	0.103
HLA-A*02:05, B*58:01, C*07:01, DRB1*03:01	30 (2.4)	0	**6.3 · 10^-4^**	**0 (0–0.6)**	**0.015**
HLA-A*30:02, B*14:02, C*08:02, DRB1*03:01	8 (0.6)	10 (2.7)	0.002	4.3 (1.5–12.8)	1
**Partial HLA haplotypes**					
HLA-A*02:05, B*58:01, C*07:01	107 (8.6)	12 (3.3)	**3.7 · 10^-4^**	**0.4 (0.2–0.7)**	**0.011**
HLA-A*02:05, B*58:01, DRB1*16:01	82 (6.6)	10 (2.7)	0.004	0.4 (0.2–0.8)	0.107
HLA-A*02:05, C*07:01, DRB1*16:01	106 (8.6)	18 (4.9)	0.025	0.6 (0.3–0.9)	0.654
HLA-B*58:01, C*07:01, DRB1*16:01	101 (8.2)	16 (4.4)	0.016	0.5 (0.3–0.9)	0.454
HLA-A*02:05, B*58:01, DRB1*03:01	31 (2.5)	0	**6.8 · 10^-4^**	**0 (0–0.4)**	**0.016**
HLA-A*02:05, C*07:01, DRB1*03:01	57 (4.6)	6 (1.6)	0.009	0.3 (0.1–0.8)	0.226
HLA-B*58:01, C*07:01, DRB1*03:01	39 (3.2)	6 (1.6)	0.150	0.5 (0.2–1.2)	1
HLA-A*02:05, B*18:01, DRB1*16:01	46 (3.7)	2 (0.5)	**6.9 · 10^-4^**	**0.1 (0.0–0.6)**	**0.017**
HLA-A*30:02, B*14:02, C*08:02	13 (1.1)	14 (3.8)	**8.2 · 10^-4^**	**3.8 (1.6–8.8)**	**0.025**
HLA-A*30:02, B*14:02, DRB1*03:01	8 (0.6)	10 (2.7)	0.002	4.3 (1.5–12.8)	1
HLA-A*30:02, C*08:02, DRB1*03:01	8 (0.6)	10 (2.7)	0.002	4.3 (1.5–12.8)	1
HLA-B*14:02, C*08:02, DRB1*03:01	22 (1.8)	10 (2.7)	0.285	1.6 (0.7–3.5)	1
HLA-A*02:05, B*58:01	109 (8.8)	12 (3.3)	**2.6 · 10^-4^**	**0.4 (0.2–0.7)**	**0.007**
HLA-A*02:05, C*07:01	209 (16.9)	36 (9.9)	**8.9 · 10^-4^**	**0.5 (0.4–0.8)**	**0.028**
HLA-A*02:05, DRB1*16:01	138 (11.1)	26 (7.1)	0.030	0.6 (0.4–1.0)	1
HLA-B*58:01, C*07:01	134 (10.8)	22 (6.0)	0.006	0.5 (0.3–0.9)	0.193
HLA-B*58:01, DRB1*16:01	102 (8.2)	16 (4.4)	0.012	0.5 (0.3–0.9)	0.347
HLA-C*07:01, DRB1*16:01	158 (12.8)	34 (9.3)	0.081	0.7 (0.5–1.0)	1
HLA-A*02:05, DRB1*03:01	111 (9.0)	22 (6.0)	0.084	0.7 (0.4–1.1)	1
HLA-B*58:01, DRB1*03:01	43 (3.5)	6 (1.6)	0.084	0.5 (0.2–1.1)	1
HLA-C*07:01, DRB1*03:01	109 (8.8)	24 (6.6)	0.196	0.7 (0.4–1.2)	1
HLA-A*30:02, B*14:02	14 (1.1)	16 (4.4)	**2.2 · 10^-4^**	4.0 (1.8–9.0)	**0.006**
HLA-A*30:02, C*08:02	13 (1.1)	14 (3.8)	**8.2 · 10^-4^**	3.8 (1.6–8.8)	**0.025**
HLA-A*30:02, DRB1*03:01	158 (12.8)	32 (8.8)	0.042	0.7 (0.4–1.0)	1
HLA-B*14:02, C*08:02	73 (5.9)	26 (7.1)	0.387	1.2 (0.7–2.0)	1
HLA-B*14:02, DRB1*03:01	22 (1.8)	10 (2.7)	0.285	1.6 (0.7–3.5)	1
HLA-C*08:02, DRB1*03:01	22 (1.8)	10 (2.7)	0.285	1.6 (0.7–3.5)	1

P_c_ was obtained by multiplying the uncorrected P value by the number of alleles observed.

Results displaying a P_c_ < 0.05 are in bold.

Conversely, the HLA-A*30:02, B*14:02, C*08:02 three-loci haplotype was overexpressed in patients compared to the controls [OR 3.8 (95% CI 1.8 –8.1), P = 0.0008, Pc = 0.025].

**Figure 1:**
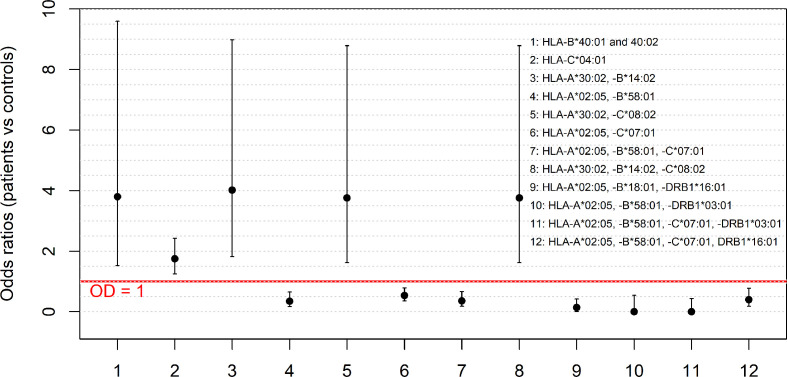
The HLA alleles and haplotypes which confer susceptibility to or protection against SARS-CoV-2 infection. The figure shows the odds ratios (OR) for the significantly different frequencies observed for HLA alleles and haplotypes (HLA-B*40:02; HLA-C*04:01; HLA-A*30:02, -B*14:02; HLA-A*02:05, -B*58:01; HLA-A*30:02, -C*08:02; HLA-A*02:05, -C*07:01; HLA-A*02:05, -B*58:01, -C*07:01; HLA-A*30:02, -B*14:02, -C*08:02; HLA-A*02:05, -B*58:01, -DRB1*03:01; HLA-A*02:05, -B*18:01, -DRB1*16:01; HLA-A*02:05, -B*58:01, -C*07:01, -DRB1*03:01 and HLA-A*02:05, -B*58:01, -C*07:01, DRB1*16:01) in the two groups of COVID-19 patients and controls. The error bars, computed according to the two-tailed Fisher’s exact test, represent the 95% confidence intervals of the OR. The red line represents the threshold OR = 1 at which the HLA allele and haplotype frequencies in the two groups of patients and controls are equal.

Overall, the analysis of the HLA alleles and haplotype frequencies revealed seven HLA alleles or haplotypes with a protective effect (OR <1) against SARS-CoV-2 infection and five alleles or haplotypes that instead were associated with an increased susceptibility to infection (**Figure 1**).

### Correlation Between HLA Allele and Haplotype Frequencies and Severity of Clinical Manifestations in SARS-CoV-2 Infection

Comparisons of the allele frequencies between the two groups A and S are shown in **Table 4** and the **Supplementary Material**. The alleles that remained significant after correction for multiple comparisons were HLA-A*23:01 and HLA-DRB1*08:01. These two alleles were exclusively present in patients with a moderate or severe disease course [OR > 2.5 (95% CI 2.7–220.6), P = 0.002, Pc = 0.038 and OR > 2.5 (95% CI 2.7–220.6), P = 0.002; Pc = 0.024, respectively]. However, it must be considered that HLA-A*23:01 is an uncommon allele in the Sardinian population for which it is difficult to assess its true impact on the evolution of the disease. Furthermore, the association with the severe form of the disease could also be distorted by the limited number of subjects who carried this allele and the fact that they all belonged to the same family nucleus.

**Table 4:** Comparison of HLA allele and haplotype frequencies between patients with pauci-symptomatic/mild (Group A) and moderate/severe disease (Group S).

Alleles/haplotypes	Healthy controls (1238 alleles)		Group A (286 alleles)		Group S (78 alleles)			
	n (%)		n (%)		n (%)	P	OR (95% CI)	P_c_
**HLA-A**								
A*23:01	20 (1.6)		0		4 (5.1)	**0.002**	> 2.5	**0.034**
A*69:01			0		2 (2.6)	**0.045**	> 0.7	0.765
**HLA-B**								
B*07:02 or B*07:05	37 (3.0)		6 (2.1)		6 (7.7)	**0.025**	3.9 (1.0–14.9)	0.950
B*40:02	11 (0.9)		6 (2.1)		6 (7.7)	**0.029**	3.9 (1.2–12.4)	0.950
**HLA-C**								
C*12:02, 12:03	77 (6.2)		34 (11.9)		2 (2.6)	**0.010**	0.2 (0.0–0.8)	0.120
**HLA-DRB1**								
DRB1*08:01	23 (1.9)		0		4 (5.1)	**0.002**	> 2.5	**0.024**
DRB1*11:01, 11:02, 11:03, 11:04	195 (15.8)		42 (14.7)		4 (5.1)	**0.022**	0.3 (0.1–0.9)	0.252
DRB1*04:03 or DRB1*04:05	164 (13.2)		32 (11.2)		16 (20.5)	**0.038**	2.0 (1.0–4.1)	0.456
**Haplotypes**								
HLA-A*02:05, B*58:01, C*07:01	107 (8.6)		12 (4.2)		0	0.077	0 (0–1.3)	1
HLA-A*30:02, B*14:02, C*08:02	13 (1.1)		4 (1.4)		10 (12.8)	**5.9 · 10^-5^**	10.3 (2.9–46.3)	**0.022**

P_c_ was obtained by multiplying the uncorrected P value by the number of alleles observed.

Results displaying a P or a P_c_ < 0.05 are in bold.

Only the HLA-A*30:02, B*14:02, C*08:02 three-loci haplotype maintained statistical significance after correction of the P values (**Supplementary Material**). This haplotype strongly correlated with disease severity [OR 10.4 (95% CI 3.2–34.1), P = 0.0007; Pc = 0.008].

## Discussion

Investigation of the SARS-CoV-2 infection confirmed that patients over 65 years of age, particularly males, with comorbidities or organ-associated pathologies are at a higher risk of developing a severe, critical or even fatal disease course (42). Consistent with the findings of other authors, type I diabetes mellitus was more common in patients with the severe clinical manifestations of SARS-CoV-2. Concomitant conditions such as hypertension and cardiovascular disease, obesity and/or a pro-inflammatory and pro-coagulative state are all likely to increase the risk of a worse outcome (43). The fact that type I diabetes mellitus was not significantly associated to a worse prognosis in our study can be attributed to the limited number of patients affected by this comorbidity and evidence of a good glucometabolic control. Overall, our findings suggest that autoimmune diseases (rheumatoid arthritis, autoimmune hepatitis, and type 1 diabetes) are associated with the most severe cases.

In autoimmune disorders, impaired regulation of cell-mediated immune response mechanisms triggers an exaggerated release of proinflammatory cytokines and chemokines by T lymphocytes leading to the so-called “cytokine storm” that often complicates the disease course of COVID-19 (5). Indeed, it appears that SARS-CoV-2 positive patients receiving immunosuppressive treatment for an autoimmune pathology have a much lower risk of developing severe clinical manifestations, and so far no complications such as acute respiratory distress syndrome (ARDS) have been reported in these patients (44, 45).

Similar to the data from Wuhan (46), Lombardy (47) and the USA (48), also in our study the female gender was associated with less severe clinical symptoms. In fact, only 28% of the patients who required oxygen therapy or mechanical ventilation were female. Female sex hormones can only partially explain this protection against SARS-CoV-2 infection. An important role can certainly be attributed to the X chromosome and, in particular, to the conservation of interacting gene clusters related to immune response (49). These genes (microRNA, TLR-7, TLR-8, the chemokine receptor CXCR3 and the interleukin-2 receptor subunit gamma IL2RG etc.) are more highly expressed in women than in males (50). Chromosome X inactivation is random in normal females for which the X chromosome inherited from the mother is active in some cells and the X chromosome inherited from the father is active in others. However, some genes on the X chromosome escape X-inactivation and may at least partially explain why women develop higher innate, humoral and cellular immune responses against pathogens and viral infections (49).

Human Leukocyte Antigen (HLA) molecules play a critical role in the battle between host innate and adaptive (cell-mediated and humoral) immune response mechanisms and invading pathogens. Epitope specificity of HLA Class I molecules creates diversity in innate immune response mechanisms and sees NK cells among the main protagonists. This lymphocyte subpopulation regulates its activity through binding of HLA class I molecules with specific activating and inhibitory receptors (killer cell immunoglobulin-like receptors, NKG2D etc.) and thereby mediates innate defense strategies against viral infections (51, 52).

The HLA complex also has a central role in the recognition and presentation of viral antigens to the immune system (CD4+ and CD8+ lymphocytes) generating a wide range of more or less effective responses. The degree of affinity of the different HLA molecules for specific viral peptides of coronaviruses, such as SARS-CoV and MERS-CoV, drastically influences immune response mechanisms and the consequential clinical manifestations of infection (28–33).

The HLA is a highly polymorphic system with profound differences in different geographical areas. For this reason, association studies in the literature often report different and sometimes conflicting results. In the study conducted by Ellinghaus and colleagues, analysis of the classical HLA loci (53) was performed to identify which variants could be correlated to the clinical variability of the COVID-19 disease course. All the HLA allelic variants, observed in 750 patients from Lombardy (northern Italy region) and 850 Spanish patients, lost significance after appropriate statistical correction.

Conversely, a study including about 500,000 voluntary donors enrolled in the Italian Bone Marrow Donor Registry (IBMDR) and representative of the entire Italian peninsula, compared the distribution of certain genes crucial to functioning of the immune system to examine whether they could underlie the geographic differences in COVID-19 incidence. According to the results, the prevalence of the HLA-C*01 and HLA-B*44 alleles are predictive of the incidence of COVID-19 in the different Italian regions and therefore confer susceptibility to SARS-CoV-2 infection (34). This association was not observed in the Sardinian population, probably because the HLA-C*01 and HLA-B*44 alleles, while being fairly common in the north eastern areas of Italy, are scarcely represented in the central-south (34). In other populations, the HLA-B*44 allele would seem to exert a protective effect (54), as opposite to what was observed by Correale et al. (34). In our study of the Sardinian population, we found that the HLA-C*04:01 allele conferred susceptibility to SARS-CoV-2 infection.

NK cell function is regulated by a large number of receptors that transmit either activating or inhibitory signals. Killer cell immunoglobulin-like receptors (KIRs) are among the most important receptors used by NK cells to distinguish viruses and other pathogens from healthy self-counterparts. HLA-C is the dominant ligand for KIR on NK cells. All HLA-C allotypes fall into two major groups of KIR epitopes (HLA-C1 and HLA-C2) on the basis of a dimorphism at position 80 of the alpha domain. The allele found associated to SARS-Cov-2 in our study encodes an HLA-C antigen pertaining to the HLA-C2 group which has high binding affinity for the activating KIR2DS1 receptor and its inhibitory homologue KIR2DL1 (55). Upon binding to HLA-C2, the inhibitory signal generated by KIR2DL1 would seem to override target cell-induced activating signals *via* KIR2DS1. It is plausible that the higher frequency of HLA-C*04:01 molecules observed in our SARS-CoV-2 positive patients creates an imbalance between HLA molecules of group C1 in favor of those of HLA-C2. This could cause a reduction in some of the functional KIR-HLA ligand interactions with a consequent decrease in the ability of NK cells to control infection.

One of the most interesting findings emerging from the analysis of HLA in Sardinian SARS-CoV-2 patients was the total absence of the extended haplotype HLA-A*02:05, B*58:01, C*07:01, DRB1*03:01. This HLA extended haplotype is third in order of frequency in the Sardinian population (36) and would therefore seem to confer protection against SARS-CoV-2 infection. The three-loci haplotype HLA-A*02:05, B*58:01 DRB1*03:01, derived from this extended haplotype, was also completely absent in SARS-CoV-2 patients whereas the other three-loci haplotype HLA-A*02:05, B*58:01 C*07:01 was present with a significantly lower frequency in patients compared to the general Sardinian population. However, patients carrying the latter three-loci haplotype only had mild disease. Therefore, it is likely that both these three-loci haplotypes exert a protective effect against the severe clinical manifestations of SARS-CoV-2 infection.

The extended haplotype HLA-A*02:05, B*58:01, C*07:01, DRB1*03:01 is extremely rare in other populations where it has a frequency of <0.1% (http://www.allelefrequencies.net/). Therefore, it will not be possible to confirm this result in other geographical areas. The Sardinian population still today represents a genetic isolate with a lowly polymorphic HLA system. This low level of polymorphism makes it easier to analyze the role of HLA molecules in the onset and progression of diseases that are strongly influenced by the immune system.

It is interesting to note how the protective effect against SARS-CoV-2 infection is provided by the extended HLA haplotype and not the individual component alleles. This suggests that combinations rather than single HLA class I and II alleles are able to generate interactions within the complex network of the immune system that allow for an efficient fight against infection.

On the contrary, we observed a greater susceptibility to SARS-CoV-2 infection and a severe disease course in subjects who possessed the HLA-A*30:02, B*14:02, C*08:02 three-loci haplotype. Previous studies have shown that the two-loci haplotype HLA-B*14:02, C*08:02 is associated with hypersensitivity reactions to non-nucleoside reverse transcriptase inhibitors (in particular nevirapine) which are used in the treatment of HIV (56), while HLA-A*30:02 is part of an extended haplotype commonly found in Sardinia (HLA-A*30:02, B*18:01, C*05:01, DRB1*03:01) that is associated with autoimmune diseases such as multiple sclerosis and autoimmune type I hepatitis (37, 57). It is likely that these HLA molecules have low binding affinity for the antigenic peptides of SARS-CoV-2, generating immune responses that cannot effectively clear the virus, but instead overreact causing self-damage.

Finally, we need to discuss the HLA-DRB1*08 (HLA-DRB1*08:01) allele since it appears to be the allele associated with the highest risk for severe clinical manifestations in Sardinian SARS-CoV-2 patients. Because this allele confers susceptibility to numerous autoimmune diseases (58), it can be postulated that the more severe clinical manifestations observed in SARS-CoV-2 patients carrying this allele are caused by altered regulation of cell-mediated immune response (5).

It is clear that the associations between HLA and susceptibility to SARS-CoV-2 infection obtained in our study may be difficult to confirm in other populations with a more polymorphic HLA system. However, they provide valuable information on the binding affinity between viral peptides and MHC class I and II antigens and can contribute to the creation of new antiviral molecules and the selection of vaccines with greater immunogenic potential.

Another surprising finding was that none of the 39 patients with severe disease had been vaccinated against the flu which is strongly recommended, particularly for people over 65 years of age. In Sardinia, the average flu vaccination rate for this category of the population is 46.2%. There is growing consensus that influenza vaccination does not increase the risk of an adverse outcome of COVID-19 but instead may provide some protection against the onset of the more severe or critical clinical manifestations (59). The pathogenesis of influenza viruses and coronaviruses depends upon their ability to dock and enter suitable human host cells. In influenza, the two major internal proteins hemagglutinin (HA) and neuraminidase (NA) mediate virus entry whereas in COVID-19 the spike (S) protein is the leading mediator of virus entry and is a primary determinant of cell tropism and pathogenesis. HA and S proteins are both heavily glycosylated which could make them susceptible to similar components of innate and adaptive immunity (60). Further explanation for a protective effect of the flu vaccine could be that the vaccine for H1N1 seems to have a protective effect on other viruses such as H3N2, thanks to a protective cross-response mediated by Toll Like Receptors through MHC II molecules such as DRB1*04:01 (27).

Another very interesting finding is the higher frequency of G6PDH enzyme deficiency in patients with severe symptoms, compared to pauci-symptomatic patients (25.6% vs 9.8%). It should be noted that the G6PDH deficiency found in 25.6% of the 39 patients with severe clinical pictures was characterized by a marked reduction (10%) in normal enzymatic activity. The importance of this finding should not be underestimated, particularly in light of the high frequency of the G6PDH^Med^ enzymatic variant in Sardinia which fluctuates between 10-20% depending on the coastal or inland areas of the island (61). Although it is rather difficult to explain how the absence of enzymatic activity can promote the onset of severe systemic manifestations in subjects affected by COVID-19, previous studies have demonstrated that virus infection induces production of reactive oxygen species (ROS) and reactive nitrogen species (RNS), which can both cause damage to proteins, DNA and cellular components of cells when antioxidant enzyme metabolism is impaired. Since G6PDH deficiency triggers a redox imbalance in the erythrocytes leading to hemolysis and tissue damage as a result of insufficient oxygen transportation, COVID-19 might increase the mortality risk of patients with this deficit (62, 63).

Another finding worthy of mention was that none of the seriously ill patients were carriers of beta-thalassemia. Two recent reports suggest that this genetic trait is likely to exert a protective effect against COVID-19 since Italian regions with a high number of beta-thalassemia carriers such as Sicily, Puglia, and Sardinia have a very low incidence of SARS-Cov-2 infections (64, 65). This hypothesis is supported by the consideration that ORF8 protein and the surface glycoproteins of SARS-Cov-2 were shown capable of binding to porphyrins. Moreover, computational proteomic analysis revealed that some SARS-CoV-2 proteins could attack the heme on the 1-ß chain of hemoglobin, resulting in the dissociation of iron to form porphyrin SARS-Cov-2 viral proteins (66).

**Figure 2:**
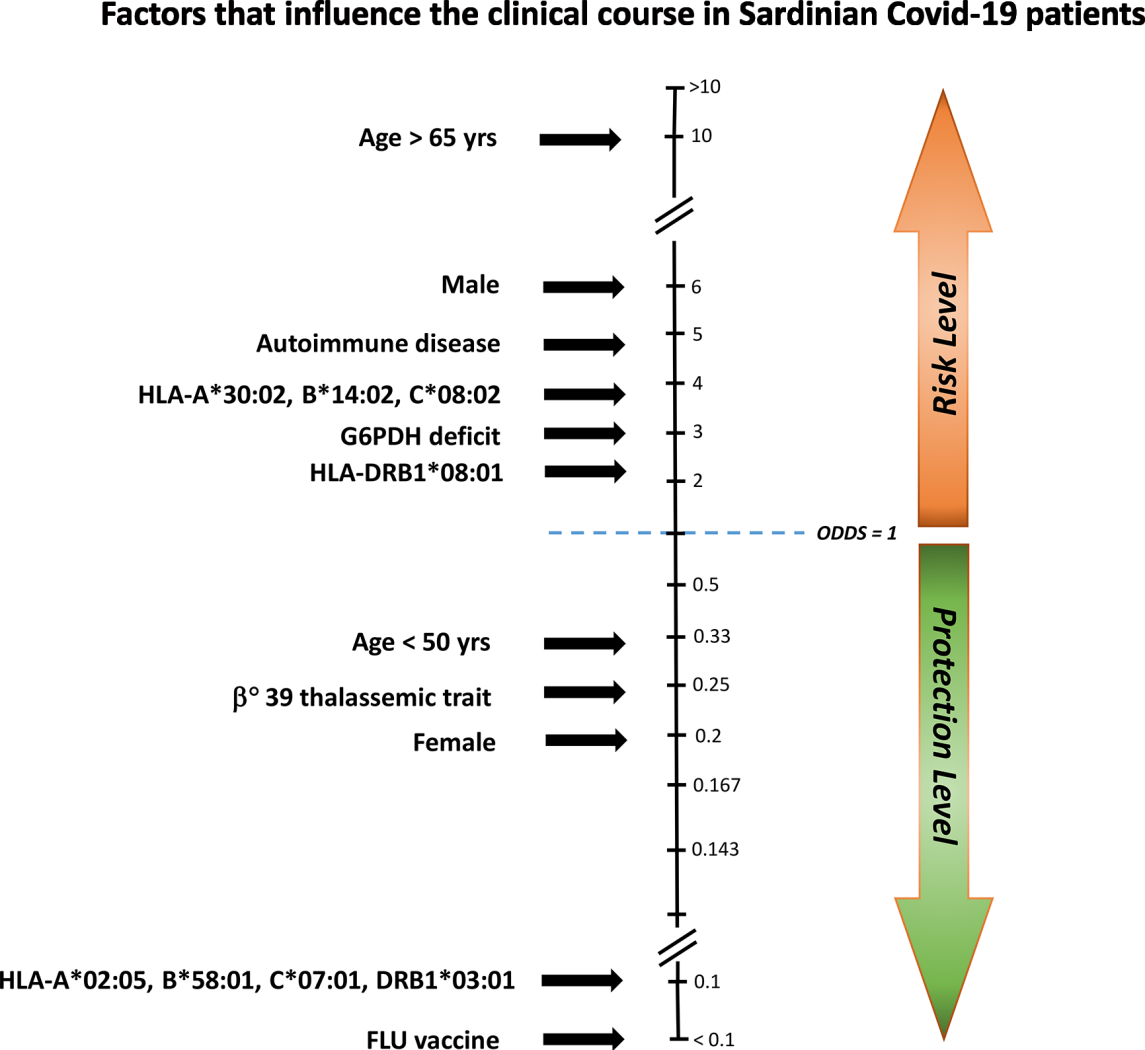
Factors influencing the clinical course of COVID-19 in Sardinian patients. To establish which factors had a major influence on the clinical disease course, we compared asymptomatic/pauci-asymptomatic patients (Group A) to those with moderate or severe symptoms (Group S). Protective and risk factors were plotted against the odds ratio (OR). Age > 65 years, male gender, autoimmune disease, the HLA-A*30:02, B*14:02, C*08:02 haplotype, G6PDH deficiency, and the HLA-DRB1*08:01 allele conferred an increased risk for severe illness, whereas influenza vaccine (FLU vaccine), the HLA-A*02:05, B*58:01, C07:01, DRB1*03:01 haplotype, female gender, and the beta(0)39-thalassemia trait would seem to offer protection.

In synthesis, this study highlights numerous clinical and genetic factors that are more or less capable of influencing the course of SARS-CoV-2, all of which can lead to the dreaded severe clinical manifestations of the infection (**Figure 2**).

The new associations with COVID-19 revealed in the present study deserve investigation on larger cohorts of patients. Meanwhile, the information provided should contribute to the optimisation of treatment and management of patients with the disease. Our results also highlight the importance of influenza vaccination in the battle against COVID-19. During the winter months, SARS-CoV-2 infection can initially easily be confused with the flu and lead to a delayed diagnosis which in turn hampers timely contact tracing procedures to contain further spread of the disease and overload of public health systems. Furthermore, this and other studies suggest that the flu vaccine may offer a certain level of protection against the more severe clinical forms of COVID-19.

However, despite the importance of the information provided, our study only represents a small step ahead in the global effort to understand the disease. Therefore, we strongly recommend analysis on larger samples and diverse populations to further unveil the immunological pathways leading to disease severity and death.

## Data Availability Statement

The raw data supporting the conclusions of this article will be made available by the authors, without undue reservation.

## Ethics Statement

The studies involving human participants were reviewed and approved by Ethics Committee of the Azienda Ospedaliero Universitaria di Cagliari. The patients/participants provided their written informed consent to participate in this study.

## Author ContributionS

RL, MC, DF, AP, and LC contributed to study conception and design. RL, MC, SD, GA, DF, SS, AL, FM, DS, WC, MK, APa, MCo, CB, GS, AP and LC especially contributed to acquiring data. RL, SC, MM, SL, SR, RS, RP, MS, PR, GO, SO, AP, LC contributed to analyzing data. RL, MC, SC, MM, AP, LC contributed to interpreting data. RL, MC, SC, MM, MGC, SDG, AR, SmD, SOr, RPe, FMa, DF, AP, and LC contributed to enhancing the intellectual content. All authors contributed to the article and approved the submitted version.

## Funding

This research was supported by: Fondazione di Sardegna: grant # 2020.2197 Associazione per l’Avanzamento della Ricerca per i Trapianti O.D.V.

## Conflict of Interest

The authors declare that the research was conducted in the absence of any commercial or financial relationships that could be construed as a potential conflict of interest.

## Abbreviations

G6PDH, glucose-6-phosphate dehydrogenase; HA, hemagglutinin; NA, neuraminidase; MOF, multiple-organ failure; ARDS, acute respiratory distress syndrome; ICU, Intensive Care Unit; SARS, Severe Acute Respiratory Syndrome; MERS, Middle East Respiratory Syndrome; HLA, human leukocyte antigen; MHC, major histocompatibility complex; TLR, toll-like receptor; RT-PCR, real time quantitative PCR.
