# Supplementary material for: Human Leukocyte Antigen Complex and Other Immunogenetic and Clinical Factors Influence Susceptibility or Protection to SARS-CoV-2 Infection and Severity of the Disease Course. The Sardinian Experience
Source: Front Immunol. 2020 Dec 4;11:605688. doi: 10.3389/fimmu.2020.605688 (PMC7746644; doi:10.3389/fimmu.2020.605688)
Supplement: Supplementary file 1 [file Table_1.docx]

**Human leukocyte antigen complex and other immunogenetic and clinical factors influence susceptibility or protection to SARS-CoV-2 infection and severity of the disease course. The Sardinian experience.**

Roberto Littera^1$*^, Marcello Campagna^2$*^, Silvia Deidda^3^, Goffredo Angioni^4^, Selene Cipri^1,5^, Maurizio Melis^2^, Davide Firinu^2^, Simonetta Santus^6^, Alberto Lai^6^, Rita Porcedda^1^, Sara Lai^1^, Stefania Rassu^1^, Rosetta Scioscia^2^, Federico Meloni^2^, Daniele Schirru^2^, William Cordeddu^3^, Marta Anna Kowalik^7^, Maria Serra^8^, Paola Ragatzu^8^, Giovanni Maria Carta^2^, Stefano Del Giacco^2^, Angelo Restivo^10^, Simona Deidda^10^, Sandro Orrù^8^, Antonella Palimodde^3^, Roberto Perra^3^, Germano Orrù^10^, Maria Conti^11^, Cinzia Balestrieri^11^, Giancarlo Serra^11^, Simona Onali^7^, Francesco Marongiu^2^, Andrea Perra^7$*^, Luchino Chessa^2,11 $*^

**HLA alleles and haplotypes in patients and controls**

The HLA allele frequencies of a cohort of 619 Sardinian controls were compared to those observed in a group of 182 patients with SARS-CoV-2 infection.

The P values corrected for multiple comparisons between controls and patients (P_c_) were obtained by multiplying the P values calculated according to the two-tailed Fisher’s exact test by the number of tested alleles (18 for HLA-A, 27 for HLA-B, 13 for HLA-C and 13 for HLA-DRB1).

The only alleles with P_c_ < 0.05 were:

HLA-B*40 [P_c_ = 0.049, OR = 3.80 (1.52 – 9.59)]

HLA-C*04 [P_c_ = 0.012, OR = 1.75 (1.25 – 2.43)].

Other alleles with P < 0.05, but with P_c_ higher than the significance threshold are highlighted in bold type in the following Tables.

| **HLA-A**  **locus** | **Controls**  **(1238 alleles)** | | **Patients**  **(364 alleles)** | | **P values** | |
| --- | --- | --- | --- | --- | --- | --- |
|  | **n** | **f (%)** | **n** | **f (%)** | **P** | **P_c_** |
| **A*11** | 81 | 6.54 | 36 | 9.89 | **0.0386** | 0.694 |
| A*80 | 0 | 0 | 2 | 0.55 | 0.0515 | 0.927 |
| A*66 | 1 | 0.08 | 2 | 0.55 | 0.131 | 1 |
| A*26 | 28 | 2.26 | 14 | 3.85 | 0.133 | 1 |
| A*30 | 230 | 18.58 | 56 | 15.38 | 0.186 | 1 |
| A*03 | 65 | 5.25 | 26 | 7.14 | 0.197 | 1 |
| A*25 | 2 | 0.16 | 2 | 0.55 | 0.224 | 1 |
| A*33 | 45 | 3.63 | 8 | 2.20 | 0.242 | 1 |
| A*29 | 26 | 2.10 | 4 | 1.10 | 0.274 | 1 |
| A*32 | 108 | 8.72 | 38 | 10.44 | 0.351 | 1 |
| A*01 | 102 | 8.24 | 24 | 6.59 | 0.375 | 1 |
| A*02 | 363 | 29.32 | 98 | 26.92 | 0.392 | 1 |
| A*74 | 5 | 0.40 | 0 | 0 | 0.594 | 1 |
| A*24 | 119 | 9.61 | 38 | 10.44 | 0.618 | 1 |
| A*23 | 20 | 1.62 | 4 | 1.10 | 0.626 | 1 |
| A*68 | 19 | 1.53 | 6 | 1.65 | 0.813 | 1 |
| A*31 | 14 | 1.13 | 4 | 1.10 | 1 | 1 |
| A*69 | 10 | 0.81 | 2 | 0.55 | 1 | 1 |

| **HLA-B**  **locus** | **Controls**  **(1238 alleles)** | | **Patients**  **(364 alleles)** | | **P values** | |
| --- | --- | --- | --- | --- | --- | --- |
|  | **n** | **f (%)** | **n** | **f (%)** | **P** | **P_c_** |
| **B*40** | 11 | 0.89 | 12 | 3.30 | **0.00183** | **0.0493** |
| **B*58** | 141 | 11.39 | 22 | 6.04 | **0.00221** | 0.0597 |
| **B*55** | 40 | 3.23 | 2 | 0.55 | **0.00247** | 0.0666 |
| **B*53** | 5 | 0.40 | 8 | 2.20 | **0.00287** | 0.0774 |
| B*56 | 3 | 0.24 | 4 | 1.10 | 0.051 | 1 |
| B*35 | 153 | 12.36 | 58 | 15.93 | 0.0784 | 1 |
| B*18 | 315 | 25.44 | 76 | 20.88 | 0.0826 | 1 |
| B*08 | 30 | 2.42 | 14 | 3.85 | 0.147 | 1 |
| B*44 | 58 | 4.68 | 24 | 6.59 | 0.175 | 1 |
| B*73 | 8 | 0.65 | 0 | 0 | 0.211 | 1 |
| B*14 | 74 | 5.98 | 28 | 7.69 | 0.271 | 1 |
| B*41 | 18 | 1.45 | 2 | 0.55 | 0.28 | 1 |
| B*45 | 18 | 1.45 | 2 | 0.55 | 0.28 | 1 |
| B*07 | 37 | 2.99 | 14 | 3.85 | 0.399 | 1 |
| B*57 | 9 | 0.73 | 4 | 1.10 | 0.507 | 1 |
| B*38 | 16 | 1.29 | 6 | 1.65 | 0.61 | 1 |
| B*78 | 4 | 0.32 | 2 | 0.55 | 0.624 | 1 |
| B*39 | 22 | 1.78 | 8 | 2.20 | 0.659 | 1 |
| B*13 | 23 | 1.86 | 8 | 2.20 | 0.667 | 1 |
| B*15 | 23 | 1.86 | 8 | 2.20 | 0.667 | 1 |
| B*49 | 75 | 6.06 | 20 | 5.49 | 0.801 | 1 |
| B*27 | 24 | 1.94 | 6 | 1.65 | 0.829 | 1 |
| B*51 | 79 | 6.38 | 22 | 6.04 | 0.903 | 1 |
| B*37 | 14 | 1.13 | 4 | 1.10 | 1 | 1 |
| B*47 | 2 | 0.16 | 0 | 0 | 1 | 1 |
| B*50 | 15 | 1.21 | 4 | 1.10 | 1 | 1 |
| B*52 | 21 | 1.70 | 6 | 1.65 | 1 | 1 |

| **HLA-C**  **locus** | **Controls**  **(1238 alleles)** | | **Patients**  **(364 alleles)** | | **P values** | |
| --- | --- | --- | --- | --- | --- | --- |
|  | **n** | **f (%)** | **n** | **f (%)** | **P** | **P_c_** |
| **C*04** | 139 | 11.23 | 66 | 18.13 | **0.000904** | **0.0118** |
| **C*07** | 369 | 29.81 | 88 | 24.18 | **0.0406** | 0.527 |
| **C*16** | 34 | 2.75 | 18 | 4.95 | **0.0436** | 0.567 |
| **C*12** | 77 | 6.22 | 34 | 9.34 | **0.0456** | 0.593 |
| C*14 | 10 | 0.81 | 0 | 0 | 0.129 | 1 |
| C*03 | 48 | 3.88 | 8 | 2.20 | 0.145 | 1 |
| C*06 | 76 | 6.14 | 16 | 4.40 | 0.249 | 1 |
| C*05 | 243 | 19.63 | 62 | 17.03 | 0.288 | 1 |
| C*08 | 73 | 5.90 | 26 | 7.14 | 0.387 | 1 |
| C*17 | 16 | 1.29 | 2 | 0.55 | 0.394 | 1 |
| C*02 | 73 | 5.90 | 24 | 6.59 | 0.618 | 1 |
| C*01 | 26 | 2.10 | 6 | 1.65 | 0.676 | 1 |
| C*15 | 54 | 4.36 | 14 | 3.85 | 0.768 | 1 |

| **HLA-DRB1**  **locus** | **Controls**  **(1238 alleles)** | | **Patients**  **(364 alleles)** | | **P values** | |
| --- | --- | --- | --- | --- | --- | --- |
|  | **n** | **f (%)** | **n** | **f (%)** | **P** | **P_c_** |
| **DRB1*14** | 34 | 2.75 | 20 | 5.49 | **0.0193** | 0.251 |
| DRB1*11 | 195 | 15.75 | 44 | 12.09 | 0.0941 | 1 |
| DRB1*12 | 19 | 1.53 | 2 | 0.55 | 0.193 | 1 |
| DRB1*07 | 68 | 5.49 | 14 | 3.85 | 0.227 | 1 |
| DRB1*13 | 51 | 4.12 | 18 | 4.95 | 0.466 | 1 |
| DRB1*08 | 23 | 1.86 | 4 | 1.10 | 0.486 | 1 |
| DRB1*16 | 240 | 19.39 | 76 | 20.88 | 0.549 | 1 |
| DRB1*01 | 104 | 8.40 | 34 | 9.34 | 0.595 | 1 |
| DRB1*04 | 164 | 13.25 | 52 | 14.29 | 0.601 | 1 |
| DRB1*03 | 272 | 21.97 | 82 | 22.53 | 0.829 | 1 |
| DRB1*09 | 2 | 0.16 | 0 | 0 | 1 | 1 |
| DRB1*10 | 23 | 1.86 | 6 | 1.65 | 1 | 1 |
| DRB1*15 | 43 | 3.47 | 12 | 3.30 | 1 | 1 |

Cochran’s rule – claiming that a minimum expected frequency of 5 can be regarded as adequate in analysing tables with more than a single degree of freedom – was used to establish which HLA haplotypes had frequencies sufficiently high to allow for significant comparisons between the two groups of patients and controls.

If $N_{i; ctr}$ and $N_{i; pts}$ are the number of times the *i*^th^ HLA haplotype is present in the groups of controls and patients, respectively, $N_{ctr}=619$ (the number of controls), $N_{pts}=182$ (the number of Covid-19 patients),$N_{tot}=N_{ctr}+N_{pts}=801$and$N_{i}= N_{i; ctr}+N_{i; pts}$. The Cochran’s rule yielded the constraint $N_{i}>5\cdot\frac{N_{tot}}{N_{pts}}=22.0$, i.e. only the HLA haplotypes present in more than 22 subjects, either Covid-19 patients or controls, were considered.

The HLA haplotypes satisfying the Cochran’s rule (with expected frequencies greater than 5) are listed in the Tables below.

For multiple comparisons between the two groups, the corrected P values (P_c_) were computed by multiplying the P values obtained according to the two-tailed Fisher’s exact test by the number of tested HLA haplotypes. P_c_ values smaller than 0.05 were only obtained for the following HLA haplotypes:

HLA-A*30, B*14 [P_c_ = 0.006, OR = 4.02 (1.82 – 8.98)]

HLA-A*02, B*58 [P_c_ = 0.007, OR = 0.35 (0.17 – 0.65)]

HLA-A*30, C*08 [P_c_ = 0.025, OR = 3.76 (1.62 – 8.79)]

HLA-A*02, C*07 [P_c_ = 0.028, OR = 0.54 (0.36 – 0.79)]

HLA-A*02, B*58, C*07 [P_c_ = 0.011, OR = 0.36 (0.18 – 0.67)]

HLA-A*30, B*14, C*08 [P_c_ = 0.025, OR = 3.76 (1.62 – 8.79)]

HLA-A*02, B*58, DRB1*03 [P_c_ = 0.016, OR = 0 (0 – 0.42)]

HLA-A*02, B*18, DRB1*16 [P_c_ = 0.017, OR = 0.14 (0.02 – 0.55)]

HLA-A*02, B*58, C*07, DRB1*03 [P_c_ = 0.015, OR = 0 (0 – 0.44)].

The second most frequent HLA four-loci haplotype HLA-A*02, B*58, C*07, DRB1*16 in the Sardinian population yielded a difference between controls and patients of P <0.05 but the corrected P value lost significance:

HLA-A*02, B*58, C*07, DRB1*16 [P = 0.004, P_c_ = 0.103, OR = 0.40 (0.18 – 0.78)].

Other HLA haplotypes with P < 0.05 but with P_c_ higher than 0.05 are highlighted in bold type in the following Tables.

| **HLA-A, -B**  **haplotypes** | | **Controls**  **(1238 alleles)** | | **Patients**  **(364 alleles)** | | **P values** | |
| --- | --- | --- | --- | --- | --- | --- | --- |
|  |  | **n** | **f (%)** | **n** | **f (%)** | **P** | **P_c_** |
| **A*30** | **B*14** | 14 | 1.13 | 16 | 4.40 | **0.000224** | **0.00606** |
| **A*02** | **B*58** | 109 | 8.80 | 12 | 3.30 | **0.000263** | **0.0071** |
| **A*03** | **B*35** | 24 | 1.94 | 18 | 4.95 | **0.00417** | 0.112 |
| **A*30** | **B*35** | 37 | 2.99 | 2 | 0.55 | **0.00576** | 0.156 |
| **A*30** | **B*18** | 174 | 14.05 | 32 | 8.79 | **0.0075** | 0.203 |
| **A*02** | **B*18** | 133 | 10.74 | 24 | 6.59 | **0.0207** | 0.559 |
| **A*02** | **B*44** | 28 | 2.26 | 16 | 4.40 | **0.0426** | 1 |
| **A*01** | **B*18** | 28 | 2.26 | 2 | 0.55 | **0.0443** | 1 |
| A*24 | B*58 | 24 | 1.94 | 2 | 0.55 | 0.0946 | 1 |
| A*30 | B*58 | 24 | 1.94 | 2 | 0.55 | 0.0946 | 1 |
| A*02 | B*35 | 56 | 4.52 | 24 | 6.59 | 0.131 | 1 |
| A*02 | B*51 | 55 | 4.44 | 10 | 2.75 | 0.174 | 1 |
| A*32 | B*35 | 26 | 2.10 | 12 | 3.30 | 0.237 | 1 |
| A*32 | B*18 | 50 | 4.04 | 20 | 5.49 | 0.244 | 1 |
| A*11 | B*35 | 37 | 2.99 | 14 | 3.85 | 0.399 | 1 |
| A*33 | B*14 | 38 | 3.07 | 8 | 2.20 | 0.476 | 1 |
| A*01 | B*58 | 22 | 1.78 | 4 | 1.10 | 0.482 | 1 |
| A*11 | B*18 | 23 | 1.86 | 4 | 1.10 | 0.486 | 1 |
| A*24 | B*18 | 45 | 3.63 | 16 | 4.40 | 0.533 | 1 |
| A*02 | B*14 | 28 | 2.26 | 10 | 2.75 | 0.561 | 1 |
| A*01 | B*08 | 21 | 1.70 | 4 | 1.10 | 0.63 | 1 |
| A*03 | B*18 | 22 | 1.78 | 8 | 2.20 | 0.659 | 1 |
| A*30 | B*51 | 19 | 1.53 | 4 | 1.10 | 0.802 | 1 |
| A*01 | B*49 | 25 | 2.02 | 6 | 1.65 | 0.829 | 1 |
| A*24 | B*35 | 31 | 2.50 | 8 | 2.20 | 0.848 | 1 |
| A*02 | B*49 | 45 | 3.63 | 14 | 3.85 | 0.874 | 1 |
| A*01 | B*35 | 21 | 1.7 | 6 | 1.65 | 1 | 1 |

| **HLA-A, -C**  **haplotypes** | | **Controls**  **(1238 alleles)** | | **Patients**  **(364 alleles)** | | **P values** | |
| --- | --- | --- | --- | --- | --- | --- | --- |
|  |  | **n** | **f (%)** | **n** | **f (%)** | **P** | **P_c_** |
| **A*30** | **C*08** | 13 | 1.05 | 14 | 3.85 | **0.000819** | **0.0254** |
| **A*02** | **C*07** | 209 | 16.88 | 36 | 9.89 | **0.000891** | **0.0276** |
| **A*03** | **C*04** | 21 | 1.70 | 16 | 4.40 | **0.00478** | 0.148 |
| **A*30** | **C*05** | 175 | 14.14 | 32 | 8.79 | **0.0075** | 0.232 |
| **A*02** | **C*04** | 50 | 4.04 | 26 | 7.14 | **0.0173** | 0.535 |
| **A*11** | **C*07** | 28 | 2.26 | 16 | 4.40 | **0.0426** | 1 |
| **A*02** | **C*02** | 48 | 3.88 | 6 | 1.65 | **0.0458** | 1 |
| A*24 | C*12 | 21 | 1.70 | 12 | 3.30 | 0.0896 | 1 |
| A*30 | C*06 | 30 | 2.42 | 4 | 1.10 | 0.149 | 1 |
| A*32 | C*02 | 19 | 1.53 | 10 | 2.75 | 0.176 | 1 |
| A*11 | C*04 | 36 | 2.91 | 16 | 4.40 | 0.178 | 1 |
| A*01 | C*07 | 70 | 5.65 | 14 | 3.85 | 0.228 | 1 |
| A*24 | C*05 | 28 | 2.26 | 12 | 3.30 | 0.256 | 1 |
| A*30 | C*04 | 35 | 2.83 | 6 | 1.65 | 0.259 | 1 |
| A*24 | C*07 | 57 | 4.60 | 12 | 3.30 | 0.308 | 1 |
| A*02 | C*16 | 17 | 1.37 | 8 | 2.20 | 0.333 | 1 |
| A*30 | C*02 | 17 | 1.37 | 8 | 2.20 | 0.333 | 1 |
| A*03 | C*07 | 34 | 2.75 | 6 | 1.65 | 0.338 | 1 |
| A*02 | C*12 | 29 | 2.34 | 12 | 3.30 | 0.344 | 1 |
| A*30 | C*07 | 63 | 5.09 | 14 | 3.85 | 0.403 | 1 |
| A*32 | C*05 | 26 | 2.10 | 10 | 2.75 | 0.428 | 1 |
| A*24 | C*04 | 32 | 2.58 | 6 | 1.65 | 0.432 | 1 |
| A*33 | C*08 | 38 | 3.07 | 8 | 2.20 | 0.476 | 1 |
| A*01 | C*04 | 21 | 1.70 | 8 | 2.20 | 0.506 | 1 |
| A*02 | C*08 | 28 | 2.26 | 10 | 2.75 | 0.561 | 1 |
| A*32 | C*07 | 58 | 4.68 | 20 | 5.49 | 0.579 | 1 |
| A*30 | C*12 | 17 | 1.37 | 6 | 1.65 | 0.625 | 1 |
| A*02 | C*06 | 28 | 2.26 | 6 | 1.65 | 0.679 | 1 |
| A*02 | C*15 | 24 | 1.94 | 8 | 2.20 | 0.831 | 1 |
| A*02 | C*05 | 89 | 7.19 | 26 | 7.14 | 1 | 1 |
| A*11 | C*05 | 21 | 1.70 | 6 | 1.65 | 1 | 1 |

| **HLA-A, -DR**  **haplotypes** | | **Controls**  **(1238 alleles)** | | **Patients**  **(364 alleles)** | | **P values** | |
| --- | --- | --- | --- | --- | --- | --- | --- |
|  |  | **n** | **f (%)** | **n** | **f (%)** | **P** | **P_c_** |
| **A*11** | **DRB1*16** | 25 | 2.02 | 18 | 4.95 | **0.00481** | 0.168 |
| **A*01** | **DRB1*16** | 29 | 2.34 | 2 | 0.55 | **0.0288** | 1 |
| **A*02** | **DRB1*16** | 138 | 11.15 | 26 | 7.14 | **0.03** | 1 |
| **A*30** | **DRB1*03** | 158 | 12.76 | 32 | 8.79 | **0.0423** | 1 |
| **A*30** | **DRB1*01** | 19 | 1.53 | 12 | 3.30 | **0.0482** | 1 |
| A*32 | DRB1*16 | 57 | 4.60 | 26 | 7.14 | 0.06 | 1 |
| A*30 | DRB1*07 | 25 | 2.02 | 2 | 0.55 | 0.0628 | 1 |
| A*02 | DRB1*03 | 111 | 8.97 | 22 | 6.04 | 0.0837 | 1 |
| A*32 | DRB1*11 | 24 | 1.94 | 2 | 0.55 | 0.0946 | 1 |
| A*32 | DRB1*01 | 18 | 1.45 | 10 | 2.75 | 0.111 | 1 |
| A*02 | DRB1*11 | 96 | 7.75 | 20 | 5.49 | 0.167 | 1 |
| A*30 | DRB1*11 | 46 | 3.72 | 8 | 2.20 | 0.187 | 1 |
| A*02 | DRB1*01 | 44 | 3.55 | 18 | 4.95 | 0.219 | 1 |
| A*24 | DRB1*04 | 22 | 1.78 | 10 | 2.75 | 0.285 | 1 |
| A*01 | DRB1*11 | 17 | 1.37 | 8 | 2.20 | 0.333 | 1 |
| A*03 | DRB1*04 | 19 | 1.53 | 8 | 2.20 | 0.362 | 1 |
| A*30 | DRB1*04 | 40 | 3.23 | 8 | 2.20 | 0.383 | 1 |
| A*03 | DRB1*11 | 25 | 2.02 | 10 | 2.75 | 0.415 | 1 |
| A*24 | DRB1*03 | 31 | 2.5 | 12 | 3.30 | 0.46 | 1 |
| A*24 | DRB1*01 | 22 | 1.78 | 4 | 1.10 | 0.482 | 1 |
| A*11 | DRB1*03 | 20 | 1.62 | 8 | 2.20 | 0.494 | 1 |
| A*11 | DRB1*04 | 21 | 1.70 | 8 | 2.20 | 0.506 | 1 |
| A*32 | DRB1*04 | 21 | 1.70 | 8 | 2.20 | 0.506 | 1 |
| A*02 | DRB1*04 | 84 | 6.79 | 28 | 7.69 | 0.559 | 1 |
| A*32 | DRB1*03 | 33 | 2.67 | 12 | 3.30 | 0.588 | 1 |
| A*02 | DRB1*15 | 17 | 1.37 | 6 | 1.65 | 0.625 | 1 |
| A*01 | DRB1*04 | 28 | 2.26 | 6 | 1.65 | 0.679 | 1 |
| A*11 | DRB1*01 | 19 | 1.53 | 4 | 1.10 | 0.802 | 1 |
| A*02 | DRB1*13 | 25 | 2.02 | 8 | 2.20 | 0.834 | 1 |
| A*24 | DRB1*16 | 38 | 3.07 | 10 | 2.75 | 0.862 | 1 |
| A*24 | DRB1*11 | 46 | 3.72 | 12 | 3.30 | 0.873 | 1 |
| A*30 | DRB1*16 | 53 | 4.28 | 16 | 4.40 | 0.884 | 1 |
| A*01 | DRB1*03 | 41 | 3.31 | 12 | 3.30 | 1 | 1 |
| A*02 | DRB1*07 | 28 | 2.26 | 8 | 2.20 | 1 | 1 |
| A*33 | DRB1*01 | 21 | 1.70 | 6 | 1.65 | 1 | 1 |

| **HLA-B, -C**  **haplotypes** | | **Controls**  **(1238 alleles)** | | **Patients**  **(364 alleles)** | | **P values** | |
| --- | --- | --- | --- | --- | --- | --- | --- |
|  |  | **n** | **f (%)** | **n** | **f (%)** | **P** | **P_c_** |
| **B*14** | **C*05** | 12 | 0.97 | 12 | 3.30 | **0.00487** | 0.146 |
| **B*58** | **C*07** | 134 | 10.82 | 22 | 6.04 | **0.00642** | 0.193 |
| **B*55** | **C*03** | 33 | 2.67 | 2 | 0.55 | **0.013** | 0.389 |
| **B*18** | **C*08** | 17 | 1.37 | 12 | 3.30 | **0.0234** | 0.702 |
| **B*18** | **C*07** | 129 | 10.42 | 24 | 6.59 | **0.0327** | 0.98 |
| **B*35** | **C*04** | 117 | 9.45 | 48 | 13.19 | **0.0493** | 1 |
| B*44 | C*04 | 15 | 1.21 | 10 | 2.75 | 0.0515 | 1 |
| B*58 | C*05 | 26 | 2.10 | 2 | 0.55 | 0.0649 | 1 |
| B*35 | C*07 | 38 | 3.07 | 18 | 4.95 | 0.103 | 1 |
| B*18 | C*05 | 214 | 17.29 | 50 | 13.74 | 0.127 | 1 |
| B*08 | C*07 | 30 | 2.42 | 14 | 3.85 | 0.147 | 1 |
| B*35 | C*05 | 31 | 2.50 | 4 | 1.10 | 0.151 | 1 |
| B*14 | C*07 | 16 | 1.29 | 8 | 2.20 | 0.221 | 1 |
| B*51 | C*07 | 27 | 2.18 | 4 | 1.10 | 0.277 | 1 |
| B*14 | C*08 | 73 | 5.90 | 26 | 7.14 | 0.387 | 1 |
| B*44 | C*05 | 32 | 2.58 | 12 | 3.30 | 0.467 | 1 |
| B*18 | C*12 | 39 | 3.15 | 8 | 2.20 | 0.479 | 1 |
| B*18 | C*06 | 20 | 1.62 | 4 | 1.10 | 0.626 | 1 |
| B*18 | C*15 | 20 | 1.62 | 4 | 1.10 | 0.626 | 1 |
| B*07 | C*07 | 26 | 2.10 | 6 | 1.65 | 0.676 | 1 |
| B*49 | C*07 | 75 | 6.06 | 20 | 5.49 | 0.801 | 1 |
| B*13 | C*06 | 19 | 1.53 | 4 | 1.10 | 0.802 | 1 |
| B*35 | C*15 | 19 | 1.53 | 4 | 1.10 | 0.802 | 1 |
| B*39 | C*07 | 19 | 1.53 | 4 | 1.10 | 0.802 | 1 |
| B*52 | C*12 | 18 | 1.45 | 6 | 1.65 | 0.807 | 1 |
| B*58 | C*04 | 18 | 1.45 | 6 | 1.65 | 0.807 | 1 |
| B*18 | C*04 | 32 | 2.58 | 8 | 2.20 | 0.849 | 1 |
| B*18 | C*02 | 21 | 1.70 | 6 | 1.65 | 1 | 1 |
| B*35 | C*12 | 20 | 1.62 | 6 | 1.65 | 1 | 1 |
| B*51 | C*02 | 35 | 2.83 | 10 | 2.75 | 1 | 1 |

| **HLA-B, -DR**  **haplotypes** | | **Controls**  **(1238 alleles)** | | **Patients**  **(364 alleles)** | | **P values** | |
| --- | --- | --- | --- | --- | --- | --- | --- |
|  |  | **n** | **f (%)** | **n** | **f (%)** | **P** | **P_c_** |
| **B*18** | **DRB1*11** | 84 | 6.79 | 10 | 2.75 | **0.00324** | 0.094 |
| **B*58** | **DRB1*16** | 102 | 8.24 | 16 | 4.40 | **0.012** | 0.347 |
| **B*35** | **DRB1*16** | 55 | 4.44 | 26 | 7.14 | **0.042** | 1 |
| B*35 | DRB1*14 | 15 | 1.21 | 10 | 2.75 | 0.0515 | 1 |
| B*14 | DRB1*16 | 16 | 1.29 | 10 | 2.75 | 0.0608 | 1 |
| B*58 | DRB1*04 | 26 | 2.10 | 2 | 0.55 | 0.0649 | 1 |
| B*58 | DRB1*03 | 43 | 3.47 | 6 | 1.65 | 0.0836 | 1 |
| B*58 | DRB1*11 | 21 | 1.70 | 2 | 0.55 | 0.134 | 1 |
| B*35 | DRB1*04 | 29 | 2.34 | 14 | 3.85 | 0.139 | 1 |
| B*18 | DRB1*04 | 48 | 3.88 | 8 | 2.20 | 0.145 | 1 |
| B*18 | DRB1*03 | 199 | 16.07 | 48 | 13.19 | 0.188 | 1 |
| B*35 | DRB1*01 | 31 | 2.50 | 14 | 3.85 | 0.205 | 1 |
| B*49 | DRB1*16 | 16 | 1.29 | 8 | 2.20 | 0.221 | 1 |
| B*08 | DRB1*03 | 22 | 1.78 | 10 | 2.75 | 0.285 | 1 |
| B*14 | DRB1*03 | 22 | 1.78 | 10 | 2.75 | 0.285 | 1 |
| B*18 | DRB1*13 | 25 | 2.02 | 4 | 1.10 | 0.37 | 1 |
| B*49 | DRB1*03 | 25 | 2.02 | 4 | 1.10 | 0.37 | 1 |
| B*51 | DRB1*11 | 41 | 3.31 | 8 | 2.20 | 0.386 | 1 |
| B*14 | DRB1*01 | 43 | 3.47 | 16 | 4.40 | 0.429 | 1 |
| B*49 | DRB1*04 | 49 | 3.96 | 18 | 4.95 | 0.456 | 1 |
| B*35 | DRB1*03 | 30 | 2.42 | 6 | 1.65 | 0.545 | 1 |
| B*18 | DRB1*01 | 27 | 2.18 | 10 | 2.75 | 0.552 | 1 |
| B*51 | DRB1*03 | 21 | 1.70 | 4 | 1.10 | 0.63 | 1 |
| B*35 | DRB1*11 | 56 | 4.52 | 14 | 3.85 | 0.663 | 1 |
| B*44 | DRB1*16 | 18 | 1.45 | 6 | 1.65 | 0.807 | 1 |
| B*51 | DRB1*04 | 24 | 1.94 | 6 | 1.65 | 0.829 | 1 |
| B*18 | DRB1*16 | 87 | 7.03 | 24 | 6.59 | 0.907 | 1 |
| B*49 | DRB1*11 | 23 | 1.86 | 6 | 1.65 | 1 | 1 |
| B*51 | DRB1*16 | 22 | 1.78 | 6 | 1.65 | 1 | 1 |

| **HLA-C, -DR**  **haplotypes** | | **Controls**  **(1238 alleles)** | | **Patients**  **(364 alleles)** | | **P values** | |
| --- | --- | --- | --- | --- | --- | --- | --- |
|  |  | **n** | **f (%)** | **n** | **f (%)** | **P** | **P_c_** |
| **C*04** | **DRB1*16** | 40 | 3.23 | 24 | 6.59 | **0.00592** | 0.19 |
| **C*04** | **DRB1*04** | 32 | 2.58 | 20 | 5.49 | **0.0105** | 0.336 |
| **C*07** | **DRB1*11** | 74 | 5.98 | 10 | 2.75 | **0.0154** | 0.492 |
| **C*05** | **DRB1*01** | 20 | 1.62 | 14 | 3.85 | **0.0204** | 0.653 |
| **C*06** | **DRB1*04** | 29 | 2.34 | 2 | 0.55 | **0.0288** | 0.922 |
| C*05 | DRB1*04 | 34 | 2.75 | 4 | 1.10 | 0.0779 | 1 |
| C*07 | DRB1*16 | 158 | 12.76 | 34 | 9.34 | 0.0814 | 1 |
| C*15 | DRB1*04 | 23 | 1.86 | 2 | 0.55 | 0.0919 | 1 |
| C*12 | DRB1*16 | 17 | 1.37 | 10 | 2.75 | 0.101 | 1 |
| C*12 | DRB1*11 | 37 | 2.99 | 16 | 4.40 | 0.185 | 1 |
| C*07 | DRB1*03 | 109 | 8.80 | 24 | 6.59 | 0.196 | 1 |
| C*08 | DRB1*16 | 16 | 1.29 | 8 | 2.20 | 0.221 | 1 |
| C*07 | DRB1*04 | 93 | 7.51 | 34 | 9.34 | 0.27 | 1 |
| C*08 | DRB1*03 | 22 | 1.78 | 10 | 2.75 | 0.285 | 1 |
| C*02 | DRB1*16 | 33 | 2.67 | 14 | 3.85 | 0.287 | 1 |
| C*05 | DRB1*11 | 42 | 3.39 | 8 | 2.20 | 0.305 | 1 |
| C*08 | DRB1*01 | 42 | 3.39 | 16 | 4.40 | 0.343 | 1 |
| C*02 | DRB1*03 | 19 | 1.53 | 8 | 2.20 | 0.362 | 1 |
| C*06 | DRB1*03 | 24 | 1.94 | 4 | 1.10 | 0.366 | 1 |
| C*06 | DRB1*07 | 24 | 1.94 | 4 | 1.10 | 0.366 | 1 |
| C*05 | DRB1*03 | 192 | 15.51 | 50 | 13.74 | 0.454 | 1 |
| C*07 | DRB1*01 | 38 | 3.07 | 8 | 2.20 | 0.476 | 1 |
| C*06 | DRB1*11 | 17 | 1.37 | 6 | 1.65 | 0.625 | 1 |
| C*12 | DRB1*03 | 17 | 1.37 | 6 | 1.65 | 0.625 | 1 |
| C*04 | DRB1*03 | 26 | 2.10 | 6 | 1.65 | 0.676 | 1 |
| C*07 | DRB1*07 | 26 | 2.10 | 6 | 1.65 | 0.676 | 1 |
| C*02 | DRB1*11 | 25 | 2.02 | 6 | 1.65 | 0.829 | 1 |
| C*07 | DRB1*13 | 24 | 1.94 | 8 | 2.20 | 0.831 | 1 |
| C*04 | DRB1*11 | 51 | 4.12 | 14 | 3.85 | 0.881 | 1 |
| C*04 | DRB1*01 | 34 | 2.75 | 10 | 2.75 | 1 | 1 |
| C*05 | DRB1*16 | 55 | 4.44 | 16 | 4.40 | 1 | 1 |
| C*15 | DRB1*16 | 21 | 1.70 | 6 | 1.65 | 1 | 1 |

| **HLA-A, -B, -C**  **haplotypes** | | | **Controls**  **(1238 alleles)** | | **Patients**  **(364 alleles)** | | **P values** | |
| --- | --- | --- | --- | --- | --- | --- | --- | --- |
|  |  |  | **n** | **f (%)** | **n** | **f (%)** | **P** | **P_c_** |
| **A*02** | **B*58** | **C*07** | 107 | 8.64 | 12 | 3.30 | **0.00037** | **0.0111** |
| **A*30** | **B*14** | **C*08** | 13 | 1.05 | 14 | 3.85 | **0.000819** | **0.0246** |
| **A*02** | **B*18** | **C*07** | 76 | 6.14 | 8 | 2.20 | **0.00193** | 0.058 |
| **A*30** | **B*35** | **C*05** | 25 | 2.02 | 0 | 0 | **0.00273** | 0.0818 |
| **A*03** | **B*35** | **C*04** | 21 | 1.70 | 16 | 4.40 | **0.00478** | 0.143 |
| **A*30** | **B*18** | **C*07** | 47 | 3.80 | 4 | 1.10 | **0.00973** | 0.292 |
| **A*30** | **B*18** | **C*05** | 171 | 13.81 | 32 | 8.79 | **0.0119** | 0.356 |
| **A*30** | **B*35** | **C*04** | 30 | 2.42 | 2 | 0.55 | **0.0301** | 0.904 |
| A*02 | B*35 | C*04 | 42 | 3.39 | 20 | 5.49 | 0.0874 | 1 |
| A*24 | B*58 | C*07 | 23 | 1.86 | 2 | 0.55 | 0.0919 | 1 |
| A*01 | B*18 | C*07 | 21 | 1.70 | 2 | 0.55 | 0.134 | 1 |
| A*30 | B*58 | C*07 | 21 | 1.70 | 2 | 0.55 | 0.134 | 1 |
| A*02 | B*51 | C*02 | 30 | 2.42 | 4 | 1.10 | 0.149 | 1 |
| A*24 | B*18 | C*07 | 20 | 1.62 | 10 | 2.75 | 0.185 | 1 |
| A*24 | B*35 | C*04 | 26 | 2.10 | 4 | 1.10 | 0.274 | 1 |
| A*11 | B*35 | C*04 | 35 | 2.83 | 14 | 3.85 | 0.304 | 1 |
| A*02 | B*44 | C*05 | 18 | 1.45 | 8 | 2.20 | 0.345 | 1 |
| A*02 | B*18 | C*05 | 79 | 6.38 | 18 | 4.95 | 0.381 | 1 |
| A*32 | B*18 | C*05 | 24 | 1.94 | 10 | 2.75 | 0.406 | 1 |
| A*32 | B*18 | C*07 | 31 | 2.50 | 12 | 3.30 | 0.46 | 1 |
| A*33 | B*14 | C*08 | 38 | 3.07 | 8 | 2.20 | 0.476 | 1 |
| A*01 | B*58 | C*07 | 22 | 1.78 | 4 | 1.10 | 0.482 | 1 |
| A*02 | B*51 | C*07 | 22 | 1.78 | 4 | 1.10 | 0.482 | 1 |
| A*02 | B*35 | C*07 | 20 | 1.62 | 8 | 2.20 | 0.494 | 1 |
| A*02 | B*14 | C*08 | 28 | 2.26 | 10 | 2.75 | 0.561 | 1 |
| A*01 | B*08 | C*07 | 21 | 1.70 | 4 | 1.10 | 0.63 | 1 |
| A*01 | B*49 | C*07 | 25 | 2.02 | 6 | 1.65 | 0.829 | 1 |
| A*24 | B*18 | C*05 | 24 | 1.94 | 8 | 2.20 | 0.831 | 1 |
| A*02 | B*49 | C*07 | 45 | 3.63 | 14 | 3.85 | 0.874 | 1 |
| A*01 | B*35 | C*04 | 20 | 1.62 | 6 | 1.65 | 1 | 1 |

| **HLA-A, -B, -DR**  **haplotypes** | | | **Controls**  **(1238 alleles)** | | **Patients**  **(364 alleles)** | | **P values** | |
| --- | --- | --- | --- | --- | --- | --- | --- | --- |
|  |  |  | **n** | **f (%)** | **n** | **f (%)** | **P** | **P_c_** |
| **A*02** | **B*58** | **DRB1*03** | 31 | 2.50 | 0 | 0 | **0.000679** | **0.0163** |
| **A*02** | **B*18** | **DRB1*16** | 46 | 3.72 | 2 | 0.55 | **0.00069** | **0.0166** |
| **A*02** | **B*58** | **DRB1*16** | 82 | 6.62 | 10 | 2.75 | **0.00447** | 0.107 |
| **A*30** | **B*18** | **DRB1*11** | 34 | 2.75 | 2 | 0.55 | **0.00861** | 0.207 |
| **A*30** | **B*18** | **DRB1*03** | 154 | 12.44 | 30 | 8.24 | **0.0311** | 0.746 |
| **A*02** | **B*18** | **DRB1*04** | 28 | 2.26 | 2 | 0.55 | **0.0443** | 1 |
| **A*32** | **B*18** | **DRB1*16** | 29 | 2.34 | 16 | 4.40 | **0.0462** | 1 |
| A*02 | B*18 | DRB1*11 | 43 | 3.47 | 6 | 1.65 | 0.0836 | 1 |
| A*02 | B*58 | DRB1*04 | 24 | 1.94 | 2 | 0.55 | 0.0946 | 1 |
| A*02 | B*51 | DRB1*11 | 29 | 2.34 | 4 | 1.10 | 0.206 | 1 |
| A*02 | B*14 | DRB1*01 | 17 | 1.37 | 8 | 2.20 | 0.333 | 1 |
| A*02 | B*35 | DRB1*16 | 30 | 2.42 | 12 | 3.30 | 0.354 | 1 |
| A*02 | B*49 | DRB1*04 | 30 | 2.42 | 12 | 3.30 | 0.354 | 1 |
| A*02 | B*18 | DRB1*03 | 76 | 6.14 | 18 | 4.95 | 0.448 | 1 |
| A*02 | B*35 | DRB1*11 | 22 | 1.78 | 4 | 1.10 | 0.482 | 1 |
| A*32 | B*18 | DRB1*03 | 27 | 2.18 | 10 | 2.75 | 0.552 | 1 |
| A*01 | B*49 | DRB1*04 | 21 | 1.70 | 4 | 1.10 | 0.63 | 1 |
| A*01 | B*08 | DRB1*03 | 19 | 1.53 | 4 | 1.10 | 0.802 | 1 |
| A*32 | B*35 | DRB1*16 | 18 | 1.45 | 6 | 1.65 | 0.807 | 1 |
| A*30 | B*18 | DRB1*16 | 39 | 3.15 | 10 | 2.75 | 0.863 | 1 |
| A*02 | B*51 | DRB1*04 | 20 | 1.62 | 6 | 1.65 | 1 | 1 |
| A*24 | B*18 | DRB1*03 | 20 | 1.62 | 6 | 1.65 | 1 | 1 |
| A*24 | B*18 | DRB1*11 | 20 | 1.62 | 6 | 1.65 | 1 | 1 |
| A*33 | B*14 | DRB1*01 | 20 | 1.62 | 6 | 1.65 | 1 | 1 |

| **HLA-A, -C, -DR**  **haplotypes** | | | **Controls**  **(1238 alleles)** | | **Patients**  **(364 alleles)** | | **P values** | |
| --- | --- | --- | --- | --- | --- | --- | --- | --- |
|  |  |  | **n** | **f (%)** | **n** | **f (%)** | **P** | **P_c_** |
| **A*11** | **C*07** | **DRB1*16** | 13 | 1.05 | 12 | 3.30 | **0.00598** | 0.156 |
| **A*30** | **C*05** | **DRB1*11** | 35 | 2.83 | 2 | 0.55 | **0.00866** | 0.225 |
| **A*02** | **C*07** | **DRB1*03** | 57 | 4.60 | 6 | 1.65 | **0.00869** | 0.226 |
| **A*30** | **C*05** | **DRB1*03** | 155 | 12.52 | 30 | 8.24 | **0.025** | 0.651 |
| **A*02** | **C*07** | **DRB1*16** | 106 | 8.56 | 18 | 4.95 | **0.0251** | 0.654 |
| A*01 | C*07 | DRB1*16 | 25 | 2.02 | 2 | 0.55 | 0.0628 | 1 |
| A*30 | C*07 | DRB1*03 | 44 | 3.55 | 6 | 1.65 | 0.0846 | 1 |
| A*02 | C*07 | DRB1*11 | 50 | 4.04 | 8 | 2.20 | 0.111 | 1 |
| A*02 | C*05 | DRB1*16 | 30 | 2.42 | 4 | 1.1 | 0.149 | 1 |
| A*32 | C*05 | DRB1*03 | 21 | 1.70 | 10 | 2.75 | 0.198 | 1 |
| A*24 | C*12 | DRB1*11 | 15 | 1.21 | 8 | 2.20 | 0.206 | 1 |
| A*24 | C*07 | DRB1*16 | 27 | 2.18 | 4 | 1.10 | 0.277 | 1 |
| A*02 | C*04 | DRB1*16 | 23 | 1.86 | 10 | 2.75 | 0.296 | 1 |
| A*02 | C*08 | DRB1*01 | 17 | 1.37 | 8 | 2.20 | 0.333 | 1 |
| A*32 | C*07 | DRB1*16 | 37 | 2.99 | 14 | 3.85 | 0.399 | 1 |
| A*02 | C*07 | DRB1*01 | 23 | 1.86 | 4 | 1.10 | 0.486 | 1 |
| A*30 | C*07 | DRB1*16 | 29 | 2.34 | 6 | 1.65 | 0.542 | 1 |
| A*02 | C*05 | DRB1*03 | 72 | 5.82 | 18 | 4.95 | 0.605 | 1 |
| A*24 | C*05 | DRB1*03 | 22 | 1.78 | 8 | 2.2 | 0.659 | 1 |
| A*02 | C*07 | DRB1*04 | 60 | 4.85 | 16 | 4.4 | 0.781 | 1 |
| A*02 | C*04 | DRB1*11 | 19 | 1.53 | 4 | 1.10 | 0.802 | 1 |
| A*30 | C*07 | DRB1*04 | 19 | 1.53 | 6 | 1.65 | 0.813 | 1 |
| A*01 | C*07 | DRB1*04 | 25 | 2.02 | 6 | 1.65 | 0.829 | 1 |
| A*01 | C*07 | DRB1*03 | 34 | 2.75 | 10 | 2.75 | 1 | 1 |
| A*30 | C*05 | DRB1*16 | 36 | 2.91 | 10 | 2.75 | 1 | 1 |
| A*33 | C*08 | DRB1*01 | 20 | 1.62 | 6 | 1.65 | 1 | 1 |

| **HLA-B, -C, -DR**  **haplotypes** | | | **Controls**  **(1238 alleles)** | | **Patients**  **(364 alleles)** | | **P values** | |
| --- | --- | --- | --- | --- | --- | --- | --- | --- |
|  |  |  | **n** | **f (%)** | **n** | **f (%)** | **P** | **P_c_** |
| **B*18** | **C*07** | **DRB1*11** | 39 | 3.15 | 2 | 0.55 | **0.00382** | 0.111 |
| **B*35** | **C*04** | **DRB1*16** | 36 | 2.91 | 22 | 6.04 | **0.00966** | 0.28 |
| **B*58** | **C*07** | **DRB1*16** | 101 | 8.16 | 16 | 4.40 | **0.0157** | 0.454 |
| **B*18** | **C*05** | **DRB1*11** | 40 | 3.23 | 4 | 1.10 | **0.0278** | 0.807 |
| B*18 | C*05 | DRB1*01 | 16 | 1.29 | 10 | 2.75 | 0.0608 | 1 |
| B*18 | C*07 | DRB1*03 | 64 | 5.17 | 10 | 2.75 | 0.0636 | 1 |
| B*18 | C*05 | DRB1*04 | 26 | 2.10 | 2 | 0.55 | 0.0649 | 1 |
| B*58 | C*07 | DRB1*04 | 26 | 2.10 | 2 | 0.55 | 0.0649 | 1 |
| B*35 | C*05 | DRB1*03 | 24 | 1.94 | 2 | 0.55 | 0.0946 | 1 |
| B*58 | C*07 | DRB1*03 | 39 | 3.15 | 6 | 1.65 | 0.15 | 1 |
| B*35 | C*04 | DRB1*04 | 24 | 1.94 | 12 | 3.30 | 0.156 | 1 |
| B*35 | C*07 | DRB1*16 | 25 | 2.02 | 12 | 3.30 | 0.165 | 1 |
| B*14 | C*08 | DRB1*16 | 16 | 1.29 | 8 | 2.20 | 0.221 | 1 |
| B*49 | C*07 | DRB1*16 | 16 | 1.29 | 8 | 2.20 | 0.221 | 1 |
| B*08 | C*07 | DRB1*03 | 22 | 1.78 | 10 | 2.75 | 0.285 | 1 |
| B*14 | C*08 | DRB1*03 | 22 | 1.78 | 10 | 2.75 | 0.285 | 1 |
| B*18 | C*07 | DRB1*16 | 57 | 4.60 | 12 | 3.30 | 0.308 | 1 |
| B*14 | C*08 | DRB1*01 | 42 | 3.39 | 16 | 4.40 | 0.343 | 1 |
| B*49 | C*07 | DRB1*03 | 25 | 2.02 | 4 | 1.10 | 0.37 | 1 |
| B*18 | C*05 | DRB1*03 | 188 | 15.19 | 48 | 13.19 | 0.4 | 1 |
| B*49 | C*07 | DRB1*04 | 49 | 3.96 | 18 | 4.95 | 0.456 | 1 |
| B*18 | C*07 | DRB1*04 | 36 | 2.91 | 8 | 2.20 | 0.585 | 1 |
| B*35 | C*04 | DRB1*03 | 21 | 1.70 | 4 | 1.10 | 0.63 | 1 |
| B*18 | C*12 | DRB1*11 | 27 | 2.18 | 6 | 1.65 | 0.676 | 1 |
| B*35 | C*04 | DRB1*01 | 29 | 2.34 | 10 | 2.75 | 0.699 | 1 |
| B*35 | C*04 | DRB1*11 | 48 | 3.88 | 12 | 3.30 | 0.753 | 1 |
| B*51 | C*02 | DRB1*11 | 19 | 1.53 | 4 | 1.10 | 0.802 | 1 |
| B*18 | C*05 | DRB1*16 | 48 | 3.88 | 14 | 3.85 | 1 | 1 |
| B*49 | C*07 | DRB1*11 | 23 | 1.86 | 6 | 1.65 | 1 | 1 |

| **HLA-A, -B, -C, -DR**  **haplotypes** | | | | **Controls**  **(1238 alleles)** | | **Patients**  **(364 alleles)** | | **P values** | |
| --- | --- | --- | --- | --- | --- | --- | --- | --- | --- |
|  |  |  |  | **n** | **f (%)** | **n** | **f (%)** | **P** | **P_c_** |
| **A*02** | **B*58** | **C*07** | **DRB1*03** | 30 | 2.42 | 0 | 0 | **0.000629** | **0.0145** |
| **A*02** | **B*58** | **C*07** | **DRB1*16** | 82 | 6.62 | 10 | 2.75 | **0.00447** | 0.103 |
| **A*30** | **B*18** | **C*05** | **DRB1*11** | 34 | 2.75 | 2 | 0.55 | **0.00861** | 0.198 |
| **A*02** | **B*18** | **C*07** | **DRB1*16** | 33 | 2.67 | 2 | 0.55 | **0.013** | 0.298 |
| **A*30** | **B*18** | **C*07** | **DRB1*03** | 42 | 3.39 | 4 | 1.10 | **0.0195** | 0.448 |
| **A*30** | **B*18** | **C*05** | **DRB1*03** | 154 | 12.44 | 30 | 8.24 | **0.0311** | 0.715 |
| A*02 | B*18 | C*07 | DRB1*11 | 26 | 2.10 | 2 | 0.55 | 0.0649 | 1 |
| A*02 | B*18 | C*05 | DRB1*16 | 24 | 1.94 | 2 | 0.55 | 0.0946 | 1 |
| A*02 | B*58 | C*07 | DRB1*04 | 24 | 1.94 | 2 | 0.55 | 0.0946 | 1 |
| A*02 | B*18 | C*07 | DRB1*04 | 22 | 1.78 | 2 | 0.55 | 0.137 | 1 |
| A*02 | B*18 | C*07 | DRB1*03 | 30 | 2.42 | 4 | 1.10 | 0.149 | 1 |
| A*02 | B*35 | C*04 | DRB1*16 | 20 | 1.62 | 10 | 2.75 | 0.185 | 1 |
| A*32 | B*18 | C*05 | DRB1*03 | 21 | 1.7 | 10 | 2.75 | 0.198 | 1 |
| A*32 | B*18 | C*07 | DRB1*16 | 22 | 1.78 | 10 | 2.75 | 0.285 | 1 |
| A*02 | B*14 | C*08 | DRB1*01 | 17 | 1.37 | 8 | 2.20 | 0.333 | 1 |
| A*02 | B*35 | C*07 | DRB1*16 | 17 | 1.37 | 8 | 2.20 | 0.333 | 1 |
| A*02 | B*49 | C*07 | DRB1*04 | 30 | 2.42 | 12 | 3.30 | 0.354 | 1 |
| A*01 | B*49 | C*07 | DRB1*04 | 21 | 1.7 | 4 | 1.10 | 0.63 | 1 |
| A*02 | B*18 | C*05 | DRB1*03 | 69 | 5.57 | 18 | 4.95 | 0.695 | 1 |
| A*01 | B*08 | C*07 | DRB1*03 | 19 | 1.53 | 4 | 1.10 | 0.802 | 1 |
| A*24 | B*18 | C*05 | DRB1*03 | 20 | 1.62 | 6 | 1.65 | 1 | 1 |
| A*30 | B*18 | C*05 | DRB1*16 | 36 | 2.91 | 10 | 2.75 | 1 | 1 |
| A*33 | B*14 | C*08 | DRB1*01 | 20 | 1.62 | 6 | 1.65 | 1 | 1 |

**HLA alleles and haplotypes in patients with mild (Group A) and severe symptoms (Group S).**

The group of 182 patients infected with SARS-CoV-2 was stratified into a group of 143 pauci-symptomatic patients or affected by mild symptoms (Group A) and a group of 39 patients with moderate or severe symptoms (Group S). The Tables in the following pages list the HLA allele frequencies in the two cohorts of patients with mild and severe symptoms. For multiple comparisons, the corrected P values (P_c_) were obtained by multiplying the P values calculated according to the two-tailed Fisher’s exact test by the number of tested alleles (17 for HLA-A, 25 for HLA-B, 12 for HLA-C and HLA-DR).

The only alleles with P_c_ < 0.05, corresponding to statistically significant comparisons, were:

HLA-A*23 (P_c_ = 0.034, OR > 2.5) and HLA-DRB1*08 (P_c_ = 0.024, OR > 2.5).

Other alleles with P < 0.05 but with P_c_ higher than the significance threshold are highlighted in bold type in the following Tables.

| **HLA-A**  **locus** | **Group A**  **(286 alleles)** | | **Group S**  **(78 alleles)** | | **P values** | |
| --- | --- | --- | --- | --- | --- | --- |
|  | **n** | **f (%)** | **n** | **f (%)** | **P** | **P_c_** |
| **A*23** | 0 | 0 | 4 | 5.1 | **0.002** | **0.034** |
| **A*69** | 0 | 0 | 2 | 2.6 | **0.045** | 0.765 |
| A*01 | 21 | 7.3 | 2 | 2.6 | 0.187 | 1 |
| A*31 | 2 | 0.7 | 2 | 2.6 | 0.202 | 1 |
| A*32 | 28 | 9.8 | 10 | 12.8 | 0.411 | 1 |
| A*11 | 32 | 11.2 | 6 | 7.7 | 0.530 | 1 |
| A*24 | 32 | 11.2 | 6 | 7.7 | 0.530 | 1 |
| A*29 | 4 | 1.4 | 0 | 0 | 0.582 | 1 |
| A*68 | 4 | 1.4 | 2 | 2.6 | 0.613 | 1 |
| A*03 | 19 | 6.6 | 4 | 5.1 | 0.795 | 1 |
| A*02 | 79 | 27.6 | 23 | 29.5 | 0.778 | 1 |
| A*25 | 2 | 0.7 | 0 | 0 | 1 | 1 |
| A*26 | 7 | 2.4 | 2 | 2.6 | 1 | 1 |
| A*30 | 45 | 15.7 | 12 | 15.4 | 1 | 1 |
| A*33 | 7 | 2.4 | 2 | 2.6 | 1 | 1 |
| A*66 | 2 | 0.7 | 0 | 0 | 1 | 1 |
| A*80 | 2 | 0.7 | 0 | 0 | 1 | 1 |

| **HLA-B**  **locus** | **Group A**  **(286 alleles)** | | **Group S**  **(78 alleles)** | | **P values** | |
| --- | --- | --- | --- | --- | --- | --- |
|  | **n** | **f (%)** | **n** | **f (%)** | **P** | **P_c_** |
| **B*07** | 7 | 2.4 | 6 | 7.7 | **0.038** | 0.950 |
| **B*40** | 7 | 2.4 | 6 | 7.7 | **0.038** | 0.950 |
| B*15 | 4 | 1.4 | 4 | 5.1 | 0.068 | 1 |
| B*53 | 4 | 1.4 | 4 | 5.1 | 0.068 | 1 |
| B*51 | 21 | 7.3 | 2 | 2.6 | 0.187 | 1 |
| B*39 | 9 | 3.1 | 0 | 0 | 0.214 | 1 |
| B*37 | 2 | 0.7 | 2 | 2.6 | 0.202 | 1 |
| B*49 | 19 | 6.6 | 2 | 2.6 | 0.271 | 1 |
| B*14 | 19 | 6.6 | 8 | 10.3 | 0.328 | 1 |
| B*38 | 7 | 2.4 | 0 | 0 | 0.354 | 1 |
| B*52 | 7 | 2.4 | 0 | 0 | 0.354 | 1 |
| B*08 | 11 | 3.8 | 4 | 5.1 | 0.537 | 1 |
| B*27 | 4 | 1.4 | 0 | 0 | 0.582 | 1 |
| B*50 | 4 | 1.4 | 0 | 0 | 0.582 | 1 |
| B*56 | 4 | 1.4 | 0 | 0 | 0.582 | 1 |
| B*57 | 4 | 1.4 | 0 | 0 | 0.582 | 1 |
| B*58 | 19 | 6.6 | 4 | 5.1 | 0.795 | 1 |
| B*18 | 58 | 20.3 | 16 | 20.5 | 1 | 1 |
| B*13 | 7 | 2.4 | 2 | 2.6 | 1 | 1 |
| B*35 | 45 | 15.7 | 12 | 15.4 | 1 | 1 |
| B*41 | 2 | 0.7 | 0 | 0 | 1 | 1 |
| B*44 | 17 | 5.9 | 4 | 5.1 | 1 | 1 |
| B*45 | 2 | 0.7 | 0 | 0 | 1 | 1 |
| B*55 | 2 | 0.7 | 0 | 0 | 1 | 1 |
| B*78 | 2 | 0.7 | 0 | 0 | 1 | 1 |

| **HLA-C**  **locus** | **Group A**  **(286 alleles)** | | **Group S**  **(78 alleles)** | | **P values** | |
| --- | --- | --- | --- | --- | --- | --- |
|  | **n** | **f (%)** | **n** | **f (%)** | **P** | **P_c_** |
| **C*12** | 34 | 11.9 | 2 | 2.6 | **0.010** | 0.120 |
| C*03 | 4 | 1.4 | 4 | 5.1 | 0.068 | 0.816 |
| C*08 | 17 | 5.9 | 8 | 10.3 | 0.206 | 1 |
| C*02 | 19 | 6.6 | 2 | 2.6 | 0.271 | 1 |
| C*05 | 45 | 15.7 | 16 | 20.5 | 0.310 | 1 |
| C*15 | 9 | 3.1 | 4 | 5.1 | 0.488 | 1 |
| C*01 | 7 | 2.4 | 0 | 0 | 0.354 | 1 |
| C*07 | 68 | 23.8 | 23 | 29.5 | 0.305 | 1 |
| C*06 | 15 | 5.2 | 2 | 2.6 | 0.544 | 1 |
| C*16 | 15 | 5.2 | 2 | 2.6 | 0.544 | 1 |
| C*04 | 51 | 17.8 | 14 | 17.9 | 1 | 1 |
| C*17 | 2 | 0.7 | 0 | 0 | 1 | 1 |

| **HLA-DRB1**  **locus** | **Group A**  **(286 alleles)** | | **Group S**  **(78 alleles)** | | **P values** | |
| --- | --- | --- | --- | --- | --- | --- |
|  | **n** | **f (%)** | **n** | **f (%)** | **P** | **P_c_** |
| **DRB1*08** | 0 | 0 | 4 | 5.1 | **0.002** | **0.024** |
| **DRB1*11** | 43 | 15.0 | 4 | 5.1 | **0.021** | 0.252 |
| **DRB1*04** | 32 | 11.2 | 16 | 20.5 | **0.038** | 0.456 |
| DRB1*01 | 23 | 8.0 | 10 | 12.8 | 0.189 | 1 |
| DRB1*10 | 7 | 2.4 | 0 | 0 | 0.354 | 1 |
| DRB1*13 | 17 | 5.9 | 2 | 2.6 | 0.387 | 1 |
| DRB1*14 | 17 | 5.9 | 2 | 2.6 | 0.387 | 1 |
| DRB1*16 | 55 | 19.2 | 18 | 23.1 | 0.523 | 1 |
| DRB1*07 | 13 | 4.5 | 2 | 2.6 | 0.747 | 1 |
| DRB1*03 | 66 | 23.1 | 16 | 20.5 | 0.760 | 1 |
| DRB1*15 | 11 | 3.8 | 2 | 2.6 | 0.743 | 1 |
| DRB1*12 | 2 | 0.7 | 0 | 0 | 1 | 1 |

The HLA haplotypes satisfying the Cochran’s rule (with expected frequencies greater than 5) are listed in the Tables below. No statistically significant differences were observed between the two groups of patients with mild or moderate and severe symptoms.

| **Two-loci HLA**  **haplotypes** | | **Group A**  **(286 alleles)** | | **Group S**  **(78 alleles)** | | **P values** | |
| --- | --- | --- | --- | --- | --- | --- | --- |
|  |  | **n** | **f (%)** | **n** | **f (%)** | **P** | **P_c_** |
| A*30 | B*18 | 23 | 8.0 | 8 | 10.3 | 0.500 | 1 |
| A*02 | B*18 | 21 | 7.3 | 4 | 5.1 | 0.619 | 1 |
| A*02 | B*35 | 19 | 6.6 | 6 | 7.7 | 0.801 | 1 |
| A*02 | C*05 | 19 | 6.6 | 8 | 10.3 | 0.328 | 1 |
| A*30 | C*05 | 23 | 8.0 | 8 | 10.3 | 0.500 | 1 |
| A*02 | C*04 | 21 | 7.3 | 6 | 7.7 | 1 | 1 |
| A*02 | C*07 | 30 | 10.5 | 8 | 10.3 | 1 | 1 |
| A*30 | DRB1*03 | 21 | 7.3 | 10 | 12.8 | 0.167 | 0.668 |
| A*02 | DRB1*04 | 19 | 6.6 | 8 | 10.3 | 0.328 | 1 |
| A*02 | DRB1*16 | 19 | 6.6 | 8 | 10.3 | 0.328 | 1 |
| A*32 | DRB1*16 | 19 | 6.6 | 6 | 7.7 | 0.801 | 1 |
| B*35 | C*04 | 40 | 14.0 | 6 | 7.7 | 0.178 | 0.534 |
| B*14 | C*08 | 17 | 5.9 | 8 | 10.3 | 0.206 | 0.618 |
| B*18 | C*05 | 36 | 12.6 | 12 | 15.4 | 0.571 | 1 |
| B*18 | DRB1*03 | 34 | 11.9 | 12 | 15.4 | 0.442 | 0.442 |
| C*07 | DRB1*04 | 23 | 8.0 | 10 | 12.8 | 0.189 | 0.567 |
| C*07 | DRB1*16 | 26 | 9.1 | 10 | 12.8 | 0.391 | 1 |
| C*05 | DRB1*03 | 36 | 12.6 | 12 | 15.4 | 0.571 | 1 |

| **Three-loci HLA**  **haplotypes** | | | **Group A**  **(286 alleles)** | | **Group S**  **(78 alleles)** | | **P values** | |
| --- | --- | --- | --- | --- | --- | --- | --- | --- |
|  |  |  | **n** | **f (%)** | **n** | **f (%)** | **P** | **P_c_** |
| A*30 | B*18 | C*05 | 23 | 8.0 | 8 | 10.3 | 0.500 | 0.500 |
| A*30 | B*18 | DRB1*03 | 21 | 7.3 | 8 | 10.3 | 0.478 | 0.478 |
| A*30 | C*05 | DRB1*03 | 21 | 7.3 | 8 | 10.3 | 0.478 | 0.478 |
| B*18 | C*05 | DRB1*03 | 34 | 11.9 | 12 | 15.4 | 0.442 | 0.442 |

| **Four-loci HLA**  **haplotypes** | | | | **Group A**  **(286 alleles)** | | **Group S**  **(78 alleles)** | | **P values** | |
| --- | --- | --- | --- | --- | --- | --- | --- | --- | --- |
|  |  |  |  | **n** | **f (%)** | **n** | **f (%)** | **P** | **P_c_** |
| A*30 | B*18 | C*05 | DRB1*03 | 21 | 7.3 | 8 | 10.3 | 0.478 | 0.478 |
